# Transcriptome analysis in different developmental stages of *Batocera horsfieldi* (Coleoptera: Cerambycidae) and comparison of candidate olfactory genes

**DOI:** 10.1371/journal.pone.0192730

**Published:** 2018-02-23

**Authors:** Hua Yang, Yan Cai, Zhihang Zhuo, Wei Yang, Chunping Yang, Jin Zhang, Yang Yang, Baoxin Wang, Fengrong Guan

**Affiliations:** 1 Key Laboratory of Ecological Forestry Engineering of Sichuan Province, College of Forestry, Sichuan Agricultural University, Chengdu, Sichuan, China; 2 Chengdu Academy of Agriculture and Forestry Sciences, Chengdu, Sichuan, China; Universita degli Studi della Basilicata, ITALY

## Abstract

The white-striped longhorn beetle *Batocera horsfieldi* (Coleoptera: Cerambycidae) is a polyphagous wood-boring pest that causes substantial damage to the lumber industry. Moreover olfactory proteins are crucial components to function in related processes, but the *B*. *horsfieldi* genome is not readily available for olfactory proteins analysis. In the present study, developmental transcriptomes of larvae from the first instar to the prepupal stage, pupae, and adults (females and males) from emergence to mating were built by RNA sequencing to establish a genetic background that may help understand olfactory genes. Approximately 199 million clean reads were obtained and assembled into 171,664 transcripts, which were classified into 23,380, 26,511, 22,393, 30,270, and 87, 732 unigenes for larvae, pupae, females, males, and combined datasets, respectively. The unigenes were annotated against NCBI’s non-redundant nucleotide and protein sequences, Swiss-Prot, Gene Ontology (GO), Pfam, Clusters of Eukaryotic Orthologous Groups (KOG), and KEGG Orthology (KO) databases. A total of 43,197 unigenes were annotated into 55 sub-categories under the three main GO categories; 25,237 unigenes were classified into 26 functional KOG categories, and 25,814 unigenes were classified into five functional KEGG Pathway categories. RSEM software identified 2,983, 3,097, 870, 2,437, 5,161, and 2,882 genes that were differentially expressed between larvae and males, larvae and pupae, larvae and females, males and females, males and pupae, and females and pupae, respectively. Among them, genes encoding seven candidate odorant binding proteins (OBPs) and three chemosensory proteins (CSPs) were identified. RT-PCR and RT-qPCR analyses showed that *BhorOBP3*, *BhorCSP2*, and *BhorOBPC1/C3/C4* were highly expressed in the antenna of males, indicating these genes may may play key roles in foraging and host-orientation in *B*. *horsfieldi*. Our results provide valuable molecular information about the olfactory system in *B*. *horsfieldi* and will help guide future functional studies on olfactory genes.

## Introduction

The white-striped longhorn beetle, *Batocera horsfieldi* (Hope) (Coleoptera: Cerambycidae), is a polyphagous wood-boring pest that feeds on more than 20 plant species belonging to taxonomically distant plant families (Salicaceae, Juglandaceae, Fagaceae, Rosaceae, Caprifoliaceae, Betulaceae, Oleaceae, Moraceae, and Euphorbiaceae) [1–5]. *B*. *horsfieldi* is distributed mainly in southwest, southern, central south, and northern China, and in Vietnam, Japan, India, and Myanmar [6]. Its basic biology is similar to other members of subfamily Lamiinae and its life cycle is usually completed in 2–3 years [7]. Adults emerge in the early summer and feed mainly on branches of host plants until they are sexually mature [8].

*B*. *horsfieldi* is a pest with a quite complete protection mechanism. The crypticity of larvae towards damage makes their prevention and control difficult and traditional chemical pest control is rarely effective. Currently, research on chemoecology and behavior has laid the foundation for exploring new prevention and control means for *B*. *horsfieldi*. Liang et al. found that *Viburnum awabuki* and *Betula luminifera* could lure *B*. *horsfieldi* adults in need of extra nutrition [4]. Li et al. found through field investigation that *B*. *horsfieldi* concentrated on rosaceous plants for extra nutrition [2]. Yang et al. found that nutrition supplement of *B*. *horsfieldi* adults was related to changes in volatile components of the plants [9]. Yang et al. analyzed encounter and mating behaviors of *B*. *horsfieldi *adults through a video capture system. The study upon encountering behavior of *B*. *horsfieldi* provides the basis for studying calling mechanism and sex pheromone biosynthesis of *B*. *horsfieldi* as well as reproductive behavior of the adults [10]. Li et al. established a cDNA library for antennae of *B*. *horsfieldi *and conducted expression analysis for relevant olfactory genes [11].

During the long evolution process, sensitive smell of insects can help them recognize external volatile chemical substances so as to realize behaviors such as searching for food, mate, and spawning places [12–14]. The main olfactory sensors of insects are the antennae at the front of the head. There are many different varieties of receptors on the antennae. The receptors contain various functional proteins related to olfactory sensation; for example, odorant binding proteins (OBPs), chemosensory proteins (CSPs), and olfactory receptors. OBPs are acidic soluble proteins with low molecular weight (about 15 kD), which are distributed mainly in lymph in the olfactory receptors of insects [15]. The typical structure of OBPs comprises six conserved cysteines, which can form three disulfide bonds to support the 3D structure of OBPs [16]. So far, OBPs have been discovered in at least seven different orders of insects, namely Lepidoptera [17–23], Diptera [24, 25], Orthoptera [26], Hemiptera [27], Isoptera [28], Hymenoptera [29], and Coleoptera [30, 31].

While OBPs have been discovered in both insects and mammals, CSPs have been found only in insects. In 1994, Mckenna et al. discovered CSPs in antennae of *Drosophila melanogaster* for the first time by subtractive hybridization [32]. The molecular weight of CSPs is lower than that of OBPs (generally only 10–15 kD). CSPs have four conserved cysteine sites [33]. The sequence similarity of CSPs is higher than that of OBPs between different insects of the same and different species [34]. So far, CSPs have been discovered in insects such as *Eurycantha calcarata* [35], *Aphis gossypii* [36], *Bombyx mori* [37], *Helicoverpa armigera* [18], *Agrotis ipsilon* [38], *Tomicus yunnanensis* [39], *Manduca sexta* [40], *Adelphocoris lineolatus* [13], and *Spodoptera littoralis* [41].

In the present study, we used RNA sequencing to identify developmental stage-specific genes by building transcriptomes of larvae from the first instar to the prepupal stage, pupae, and adults (females and males) from emergence to mating (3-day-old). We identified differentially expressed genes among larvae, pupae, and female and male adults by comparative transcriptome analysis. We also screened *B*. *horsfieldi* candidate olfactory genes, including those encoding OBPs and CSPs, because the olfactory system is crucial for insects to locate hosts, oviposition sites, and food sources. Finally, we validated the differentially expressed candidate olfactory genes identified in the transcriptome data by RT-PCR and RT-qPCR.

## Material and methods

### Insect rearing and sample collection

Larvae, pupae and adults of *B*. *horsfieldi* were collected in June 2016 from in the Poplar Planting Base of Luojiang City, Sichuan Province, China (31.07°N, 104.08°E). The field studies did not involve endangered or protected species, and no specific permission was required for the research activity at this location. Adults were used just emergence and unmated. The characteristics used to identify mated *B*. *horsfieldi* were the villi on the abdomen of mated males and the obvious mating plaques on the backside of the mated females [42]. Female and male adults were placed on ice and quickly dissected into antenna, thorax (without thoracic legs), hind wing, and thoracic legs for RT-PCR analysis. RT-qPCR was performed using nucleic acids from male and female adult organism. All samples were immediately frozen in liquid nitrogen and stored at −80°C until use. Each sample contained either larvae, pupae, or male or female adult tissues from at least five insects. After pooling the tissues for each sample, three biological replicates were conducted for each treatment.

### RNA extraction and sequencing

Mixed larvae from the first instar to the prepupal stage, pupae, and adults (females and males) were prepared for RNA extraction. Total RNA was isolated from homogenized sample in TRIzol reagent (Takara, Dalian, Liaoning, China) following the manufacturer’s protocols. The concentration of total RNA was quantified with a Qubit3.0 (Thermo Fisher Scientific, Waltham, MA, USA) and an Agilent2100 Bioanalyzer (Agilent Technologies, Santa Clara, CA, USA). UV absorption values at 260 nm/280 nm was recorded to monitor the purity of the RNA products (Nanodrop2000, Thermo Fisher Scientific, Waltham, MA, USA). After RNA extraction, mRNAs were purified using the interaction of the poly (A) tails and magnetic oligo (dT) beads and collected using RNeasy RNA reagent. Mixed mRNAs were fragmented into 300–800 bp pieces using RNA fragment reagent (Illumina), and the pieces were collected using an RNeasy RNA cleaning kit (Qiagen). Subsequently, RNA fragments were copied to make first-strand cDNA using MMLV reverse transcriptase (Takara, Dalian, Liaoning, China) and random primers. Second-strand cDNA synthesis was performed using DNA Polymerase I and RNase H. The Illumina HiSeq2000 system and 125 paired-end reads were used for sequencing. Statistical analysis of the sequence lengths was performed to ensure sequence purity.

### Assembly and functional annotation

Raw sequence data in fasta format were first processed through in-house Perl scripts [43]. In this step, clean data (clean reads) were obtained by removing reads containing adapter, poly-N, and low-quality reads from the raw sequence data [44, 45]. The Q20, Q30, GC content, and sequence duplication level of the clean data were calculated [44]. All downstream analyses were based on good-quality clean data.

The flow chart of transcriptome assembly described by Grabherr et al. [46] was used in the present analyses. A Perl pipeline described by Haas et al. [43] was used to analyze the sequence data. As suggested by Haas et al.[43], when multiple sequencing runs are conducted for a single experiment, the resultant reads can be concatenated into two files if paired-end sequencing is used. The left files (read 1 files) from all the samples were pooled into a single large left.fq file, and the right files (read 2 files) were pooled into a single large right.fq file. Transcriptome assembly was accomplished based on the left.fq and right.fq using Trinity (http://trinityrnaseq.github.io) with min_kmer_cov set to two by default and all other parameters set to default. The assembled unigenes were annotated by BLASTX searches and ESTScan against the NCBI non-redundant protein sequences (Nr), NCBI non-redundant nucleotide sequences (Nt), Swiss-Prot, Gene Ontology (GO), protein families (Pfam), Clusters of Eukaryotic Orthologous Groups (KOG), and KEGG Orthology (KO) databases (E <10^−5^), and the best annotations were selected [44, 45, 47]. Differentially expressed genes were selected based on a log^2^ fold change >1 and q value <0.005 using DESeq [48] (S1 Table). The simple sequence repeats (SSR) in the *B*. *horsfieldi* unigene sequences were screened with MISA software (http://pgrc.ipk-gatersleben.de/misa/misa.html). The expression level of genes were calculated based on FPKM method[49]. The nucleotide sequences of the identified olfactory gene are listed in S2 Table.

### Homology analysis

A neighbor-joining (NJ) tree was constructed with MEGA version 5.0 and the Jones-Taylor-Thornton model [50]. The olfactory genes of other coleopteran species were obtained from the NCBI databases. Bootstrap support values were based on 1000 replicates. All the candidate olfactory genes were named according to the nomenclature system described previously [51, 52]. The olfactory genes from different species were marked with different colors and the phylogenetic tree was generated with iTOL software (http://itol.embl.de)

### RT-PCR and RT-qPCR validation of differentially expressed candidate olfactory proteins

Seven OBPs and three CSPs that were predicted to be highly abundant in antenna or had complete ORFs were selected for further analysis. Specific primer pairs were derived from the transcriptome data, and primer pairs for each gene were designed to amplify 100–200 bp products, which were verified by sequencing. A semi-quantitative RT-PCR (Bio-Rad S1000, US) analysis was performed for each primer pair using r*Taq* DNA polymerase (Takara, Dalian, Liaoning, China) before the RT-qPCR analysis to ensure that the correct products were amplified and no primer dimers were present [53]. The RT-qPCR analysis was carried out using an Mx 3000P detection system (Agilent, Palo Alto, CA, USA) as described previously, with thermal cycler parameters of 2 min at 94°C, then 40 cycles of 20 s at 94°C, 20 s at 58°C, and 20 s at 72°C. The *18S* gene was used as an internal control: *18S* forward and reverse, 5’- GAGACTCTAGCCTGCTAACT-3’ and 5’-TGTTTGTACGCCGACAGT-3’. A standard curve was derived from 10-fold serial dilutions of plasmid containing the target DNA segment to determine the PCR efficiency and to quantify the amount of target mRNA. All primers tested gave amplification efficiencies of 90–100%. For each treatment, three biological replicates were conducted. RT-qPCR data were analyzed by the 2^−ΔΔCT^ method [54]. The primers used in this experiment were designed with Primer premier 5.0 and Oligo 6.0 and are listed in S3 Table. A Chi-square test was using to compare the expression level of male and female adult. The RT-qPCR data were analyzed and output as PDF files using Graphpad 5.0.

## Results

### Illumina sequencing and assembly

This filtering resulted in a total of 50,028,651, 51,705,759, 49,935,243, and 47,402,329 clean reads in larvae, pupae, and females and males of *B*. *horsfieldi*, respectively. All the clean reads were assembled into transcripts by Trinity software; the longest copy of redundant transcripts was regarded as a unigene [43, 44, 46]. A total of 171,664 transcripts were obtained and assembled into 87,732 unigenes. Many unigenes exceeded 2000 bp in length, while approximately 21.13% unigenes exceeded 1000 bp, and 25.74% were 500–1000 bp (Table 1).

**Table 1:** Number and length of transcripts and unigenes.

	Larval	Pupal	Female	Male
Raw reads	51,907,500	53,393,179	51,993,763	49,026,747
Clean reads	50,028,651	51,705,759	49,935,243	47,402,329
Clean bases	7.48G	7.76G	7.49G	7.11G
Q20%	96.49	96.88	96.74	95.78
Q30%	91.29	92.04	91.8	89.35
GC%	42.88	44.99	42.35	40.24
	Transcripts		Unigenes	
200–500 bp	98,529		16,827	
500–1 k bp	24,766		22,585	
1 k-2 k bp	18,588		18,539	
>2 k bp	29,781		29,781	
Total number	171,664		87,732	
Min length	201		201	
Mean length	1,188		2048	
Max length	27,920		27,920	
N50	3,143		3669	
N90	360		855	
Total nucleotides	203,893,683		179,705,476	
	Number of Unigenes		Percentage%	
Annotated in NR	50,968		58.09	
Annotated in NT	17,863		20.36	
Annotated in KO	25,814		29.42	
Annotated in SwissProt	40,700		46.39	
Annotated in PFAM	42,320		48.23	
Annotated in GO	43,197		49.23	
Annotated in KOG	25,237		28.76	
Annotated in all databases	8,275		9.43	
Annotated in at least one databases	56,507		64.4	

### Annotation of the *B*. *horsfieldi* unigenes

The assembled unigenes were annotated against the Nr, Nt, Swiss-Prot, Pfam, GO, KOG/COG, and KO databases[53]. A total of 26,511 unigenes were annotated in *B*. *horsfieldi* pupae, 23,380 in larvae, 30,270 in males, and 22,393 in females. Among them, 549 were BP-specific, 595 were BL-specific, 515 were BF-specific, 922 were BM-specific, 11,012 were common among the groups, and 87,732 were in the BP-BL-BF-BM combined dataset (Table 2). The numbers and percentages of unigenes annotated in each of the databases were counted. The Nr database had the best matches against the unigenes in the BP-BL-BF-BM combined dataset (50,968, 58.10%) (Table 2) (S1–S12 Texts).

**Table 2:** Unigenes annotated in different databases.

	BP		BL		BM		BF		BP-specific	
	NO.	PCT(%)	NO.	PCT(%)	NO.	PCT(%)	NO.	PCT(%)	NO.	PCT(%)
NR	24906	93.95%	22026	94.21%	28423	93.90%	21043	93.97%	461	83.97%
NT	8718	32.88%	7735	33.08%	8877	29.33%	7595	33.92%	160	29.14%
KO	13092	49.38%	11859	50.72%	15030	49.65%	11654	52.04%	180	32.79%
Swissprot	20419	77.02%	18296	78.25%	23089	76.28%	17736	79.20%	329	59.93%
PFAM	20415	77.01%	18186	77.78%	23183	76.59%	17535	78.31%	389	70.86%
GO	20801	78.46%	17211	73.61%	23586	77.92%	18525	82.73%	394	71.77%
KOG	13798	52.05%	11991	51.29%	15260	50.41%	11823	52.80%	176	32.06%
Total NO.	26511		23380		30270		22393		549	
	BL-specific		BM-specific		BF-specific		Common		BP-BL-BM-BF combined	
	NO.	PCT(%)	NO.	PCT(%)	NO.	PCT(%)	NO.	PCT(%)	NO.	PCT(%)
NR	476	80.00%	784	85.03%	397	77.09%	10705	97.21%	50968	58.10%
NT	360	60.50%	183	19.85%	272	52.82%	3810	34.60%	17863	20.36%
KO	271	45.55%	342	37.09%	218	42.33%	6121	55.84%	25806	29.41%
Swissprot	431	72.44%	563	61.06%	360	69.90%	9223	83.75%	40700	46.39%
PFAM	421	70.76%	660	71.58%	353	68.54%	8804	79.95%	42320	48.24%
GO	435	73.11%	563	61.06%	360	69.90%	8960	81.37%	44535	50.76%
KOG	225	37.82%	326	35.36%	174	33.79%	6528	59.28%	25237	28.77%
Total NO.	595		922		515		11012		87732	

BP: Unigenes of *Batocera horsfieldi* pupae; BL: Unigenes of *B*. *horsfieldi* larvae; BM: Unigenes of *B*. *horsfieldi* males; BF: Unigenes of *B*. *horsfieldi* females; BP-specific: Specific unigenes of *B*. *horsfieldi* pupae; BL-specific: Specific unigenes of *B*. *horsfieldi* larvae; BM-specific: Specific unigenes of *B*. *horsfieldi* males; BF-specific: Specific unigenes of *B*. *horsfieldi* females; Common: Common unigenes of *B*. *horsfieldi* pupae, larvae, males, and females; BP-BL-BM-BF Combined: Total unigenes of *B*. *horsfieldi* pupae, larvae, males, and females. NO: number; PCT (%): percentage (%); NR: NCBI non-redundant protein sequences; NT: NCBI non-redundant nucleotide sequences; KO: KEGG Orthology; Swissprot: A manually annotated and reviewed protein sequence database; PFAM: Protein family; GO Gene Ontology; KOG: Clusters of Orthologous Groups of protein; Total NO: Total number of annotated unigenes.

**Fig 1:**
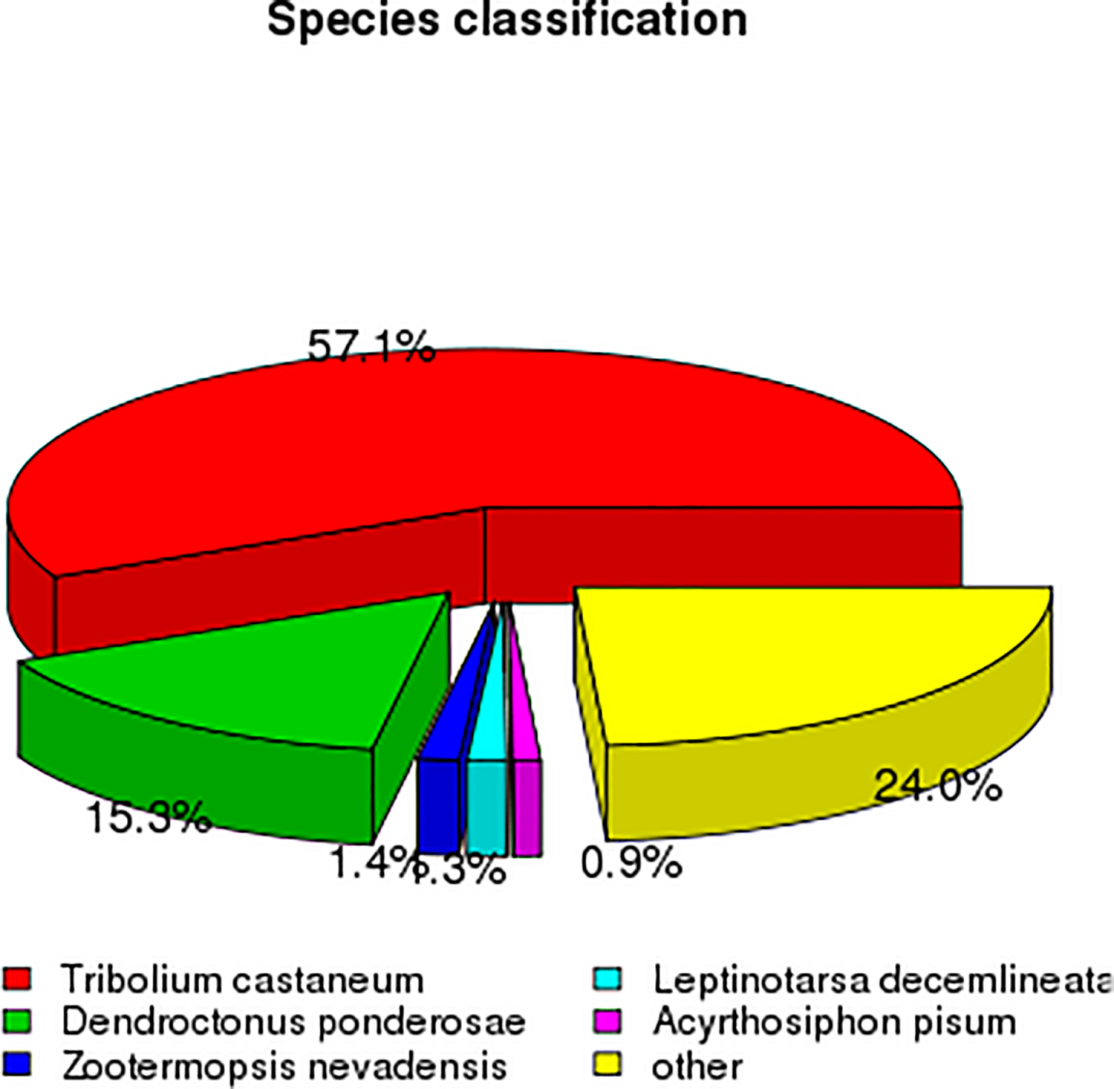
The five most represented species among the annotation results of the *B*. *horsfieldi* unigenes.

After functional annotation, the numbers of sequences from different species that matched the *B*. *horsfieldi* unigenes were calculated from the annotation results. The five most represented species (about 76% of all the species) were *Tribolium castaneum* (57.1% of the annotated sequences), *Dendroctonus ponderosae* (15.3%), *Zootermopsis nevadensis* (1.4%), *Leptinotarsa decemlineata* (1.3%), and *Acyrthosiphon pisum* (0.9%), as shown in Fig 1.

### Functional annotation of the *B*. *horsfieldi* unigenes

**Fig 2:**
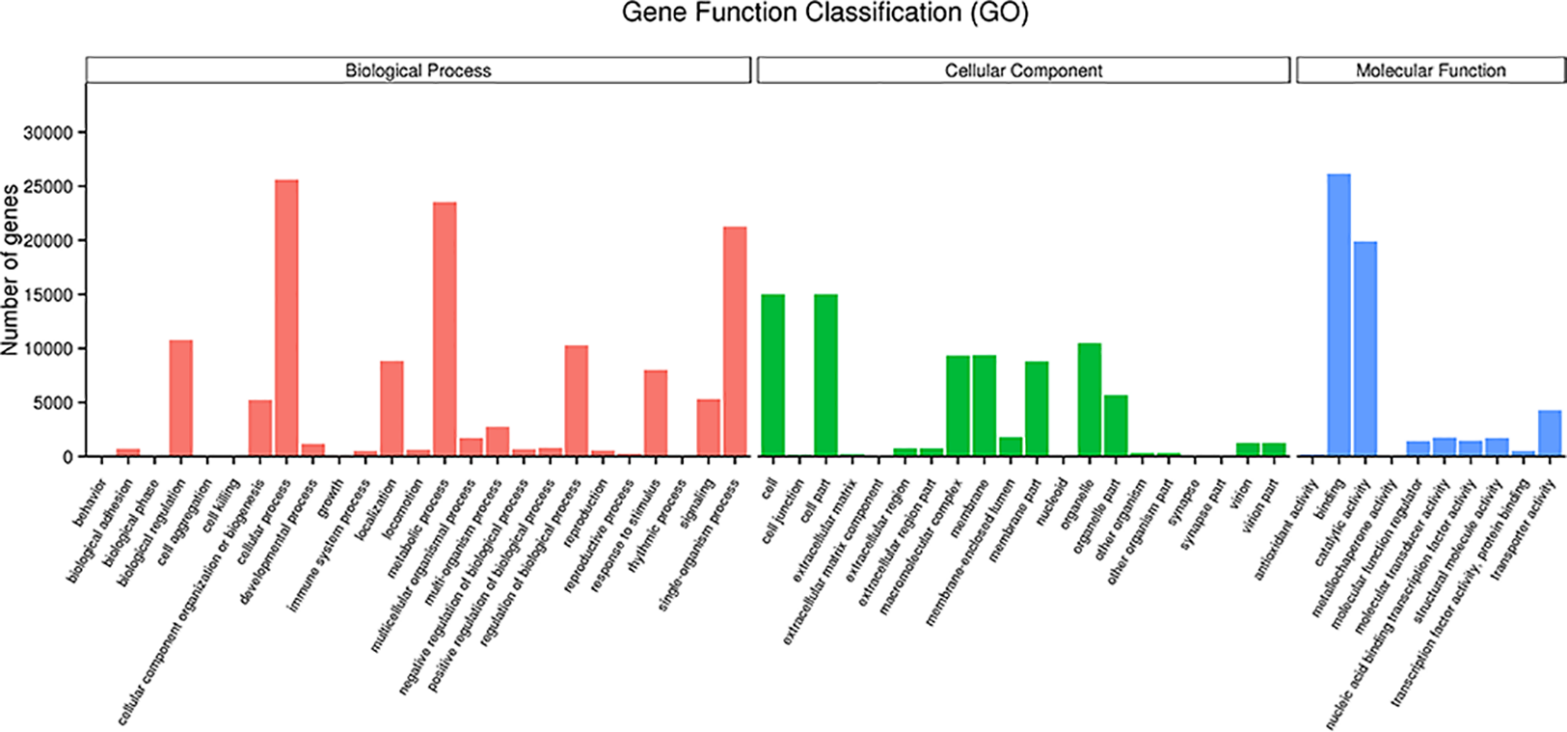
Histogram of the gene ontology (GO) classification of the *B*. *horsfieldi* unigenes.

A total of 43,197 unigenes were annotated into 55 sub-categories under the three main GO categories: biological process, cellular component, and molecular function (Fig 2). There were 25 sub-categories under biological process, 20 under cellular component, and 10 under molecular function. The top 10 sub-categories were binding (26,156 unigenes), cellular process (25,616 unigenes), metabolic process (23,538 unigenes), single-organism process (21,263 unigenes), catalytic activity (19,882 unigenes), cell (14,998 unigenes), cell part (14,998 unigenes), biological regulation (10,787 unigenes), organelle (10,503 unigenes), and regulation of biological process (10,283 unigenes) (S13 Text).

**Fig 3:**
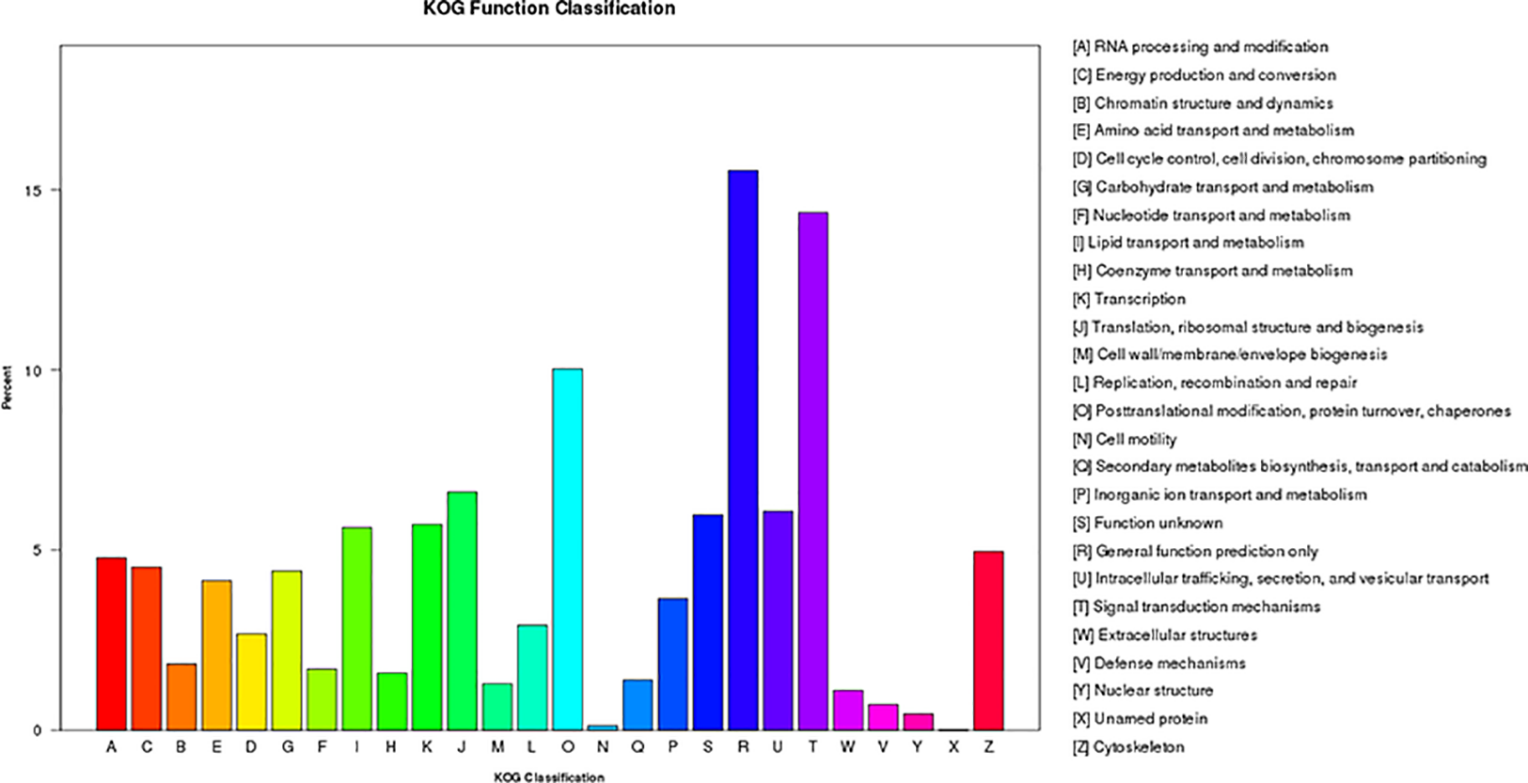
Histogram of the clusters of eukaryotic orthologous groups (KOG) classification of the *B*. *horsfieldi* unigenes.

The KOG classification placed 25,237 unigenes into 26 functional categories (Fig 3). The ‘general function prediction only’ category was the largest (3,920 unigenes), followed by ‘signal transduction mechanisms’ (3,625 unigenes), and ‘posttranslational modification, protein turnover, chaperons’ (2,531 unigenes). The top three categories had 39.93% of the unigenes assigned to KOG categories (S14 Text).

**Fig 4:**
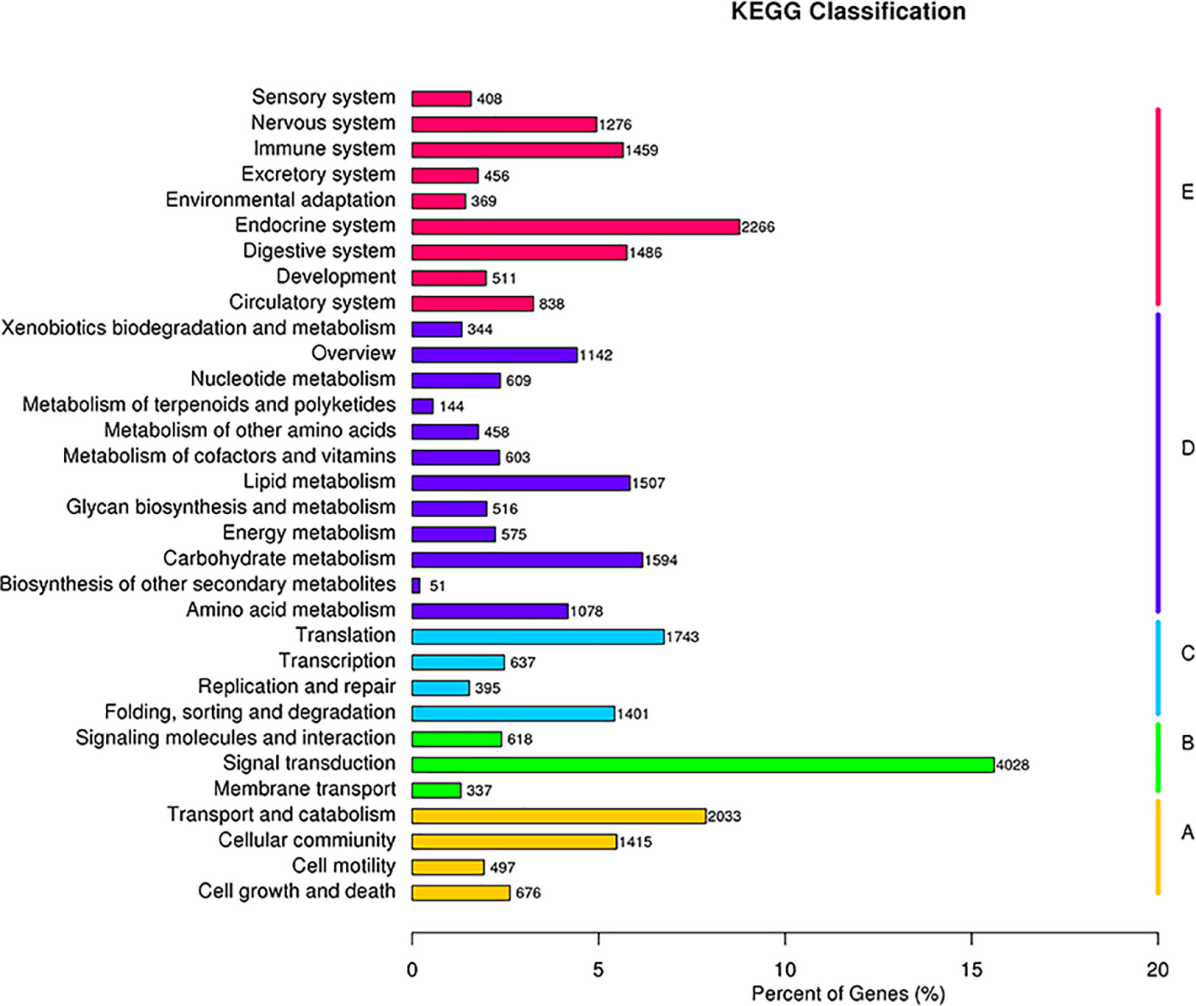
Histogram of the KEGG Pathway classification of the *B*. *horsfieldi* unigenes. (A) Cellular processes, (B) Environmental information processing, (C) Genetic information processing, (D) Metabolism, (E) Organismal systems.

A total of 25,814 unigenes were classified into five KEGG Pathway functional categories (Fig 4): cellular process (4,621 unigenes), environmental information processing (4,983 unigenes), genetic information processing (4,176 unigenes), metabolism (8,621 unigenes), and organismal system (9,069 unigenes). The top three subcategories out of a total of 32 were ‘signal transduction’, ‘endocrine system’, and ‘transport and catabolism’ (S15 Text).

### SSR analysis

**Fig 5:**
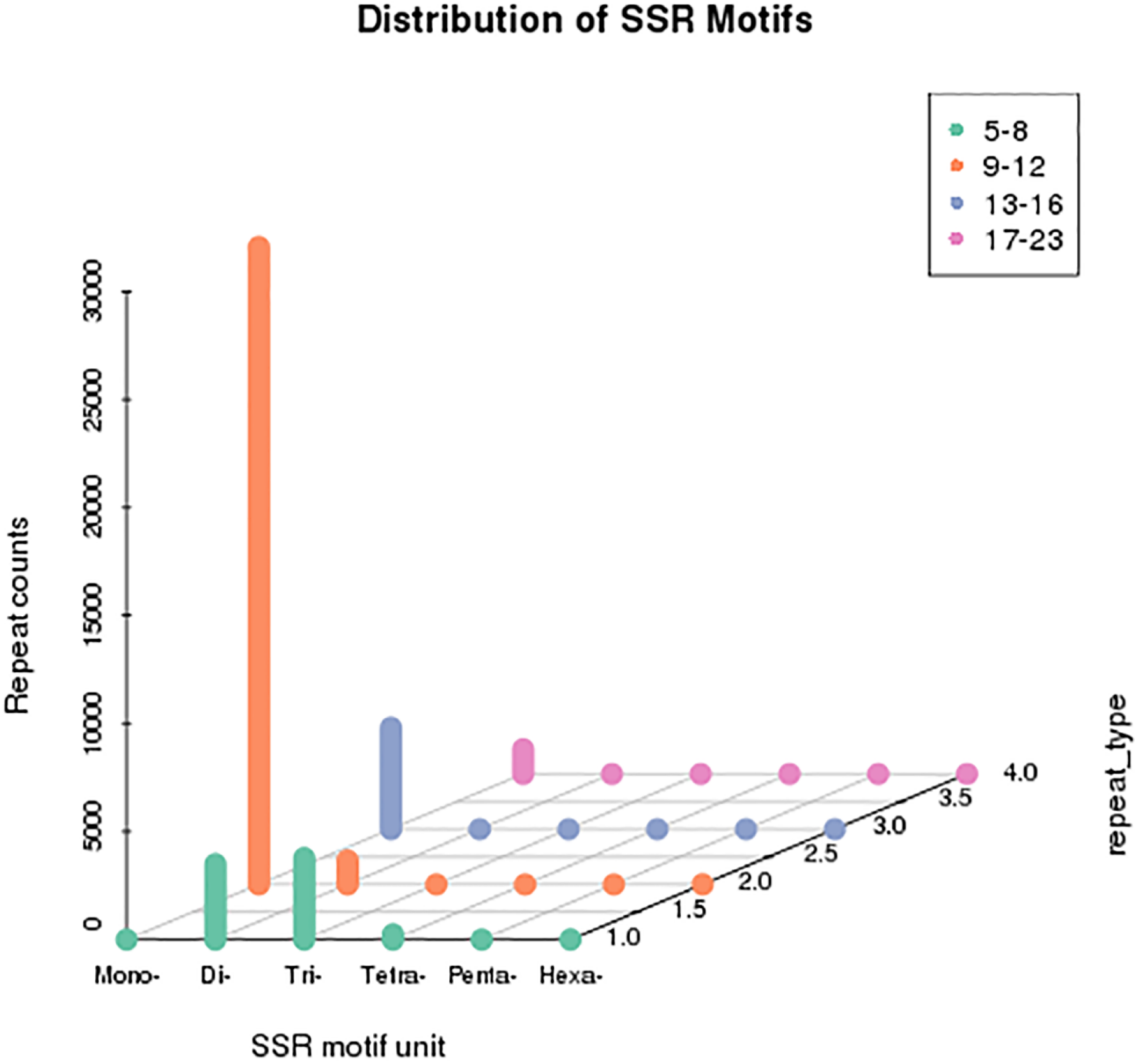
Scattergram of simple sequence repeats (SSRs) detected in the *B*. *horsfieldi* unigene sequences.

We screened for SSRs in the *B*. *horsfieldi* unigene sequences using MISA software and designed primers with Primer 3 for the SSR markers (Fig 5). We identified 87,732 sequence segments with total length of 179,705,476 bp, among which 44,015 SSRs sequences were authenticated.

### Expected number of fragments per kilobase of transcript sequence per millions base pairs sequenced (FPKM) analysis

**Fig 6:**
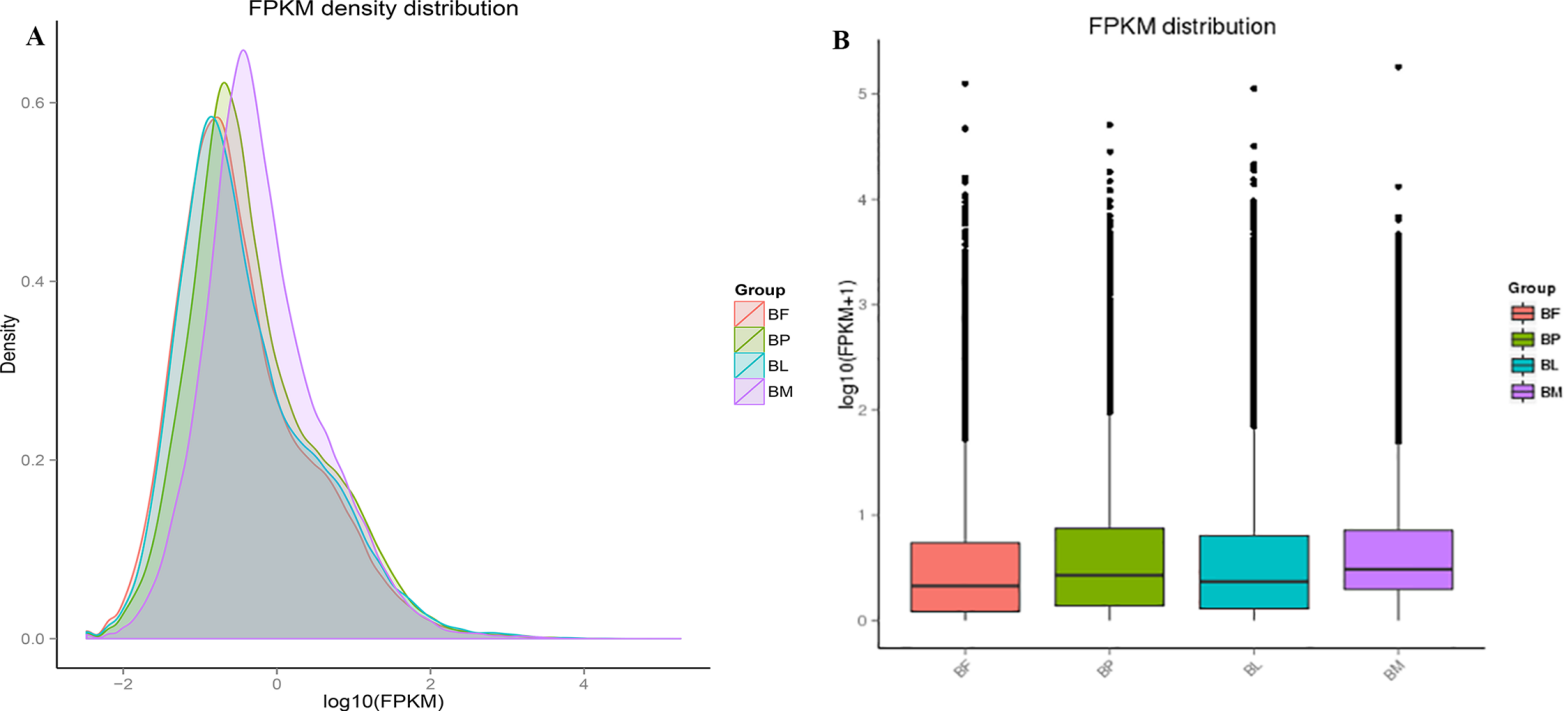
Expression levels of the *B*. *horsfieldi* unigenes by FPKM analysis. (A) The perspective of general distribution. (B) The dispersing perspective. BF: *B*. *horsfieldi* females, BP: *B*. *horsfieldi* pupae, BL: *B*. *horsfieldi* larvae, BM: *B*. *horsfieldi* males.

We used the RSEM software to calculate statistics for the bowtie comparison results, and convert FPKM[55]. We obtained the number of read counts for each gene and conducted FPKM analysis accordingly. From the perspective of general distribution of expression quantity (Fig 6A) and discrete angle (Fig 6B), the gene expression quantity of different forms of *B*. *horsfieldi* are different.

### Differentially expressed genes

**Fig 7:**
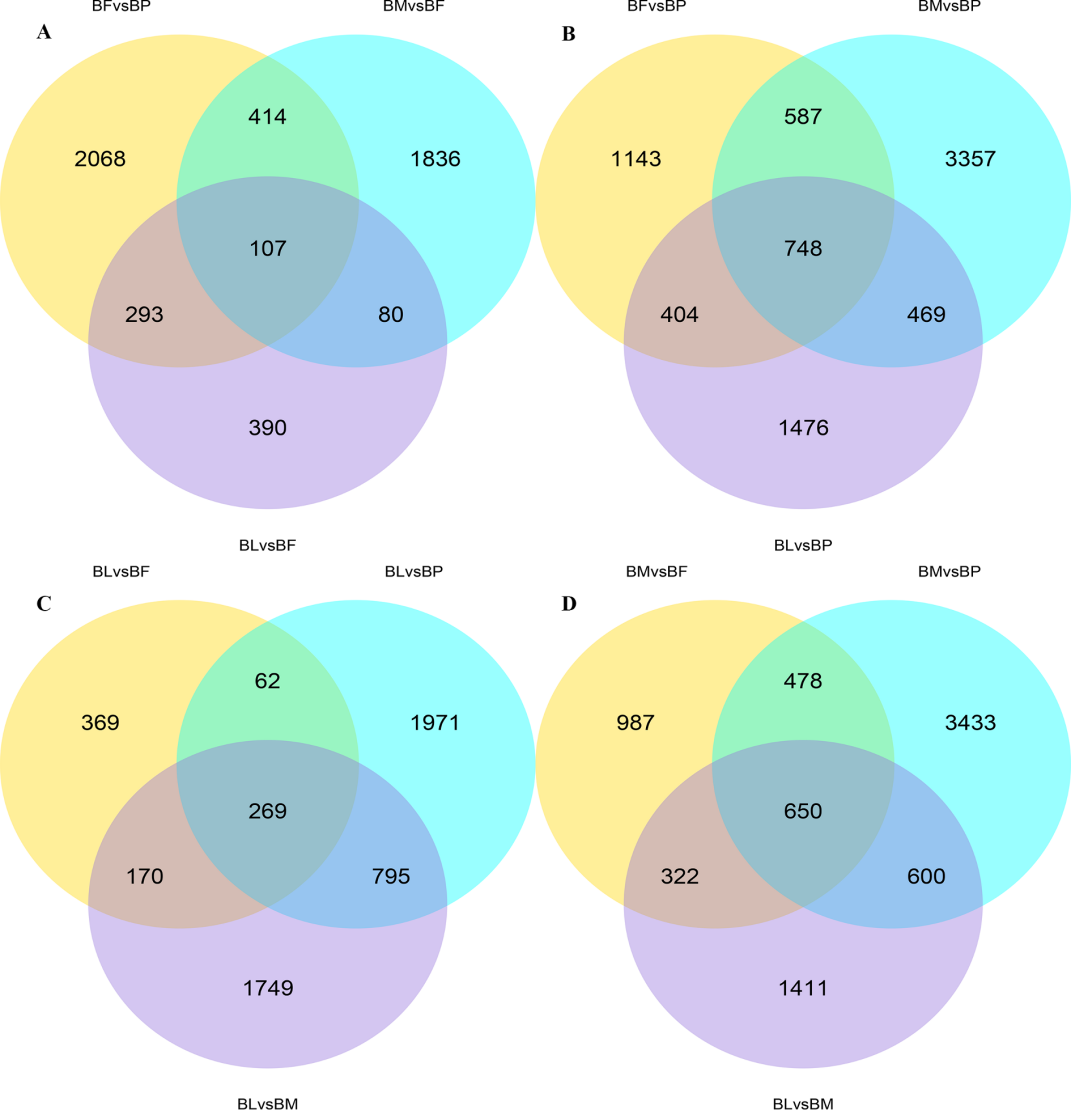
Venn diagram of the number of differentially expressed genes in males, females, larvae, and pupae. BF: *B*. *horsfieldi* females, BP: *B*. *horsfieldi* pupae, BL: *B*. *horsfieldi* larvae, BM: *B*. *horsfieldi* males.

A total of 2,882, 2,437, and 870 genes were differentially expressed between female and pupae, female and male, female and larvae, respectively, and 107 of these genes were common to pupae, males, larvae, and females (Fig 7A). A total of 2,882, 5,161, and 3,097 genes were differentially expressed between pupae and females, pupae and males, pupae and larvae, respectively, and 748 of these genes were common to males, females, larvae, and pupae (Fig 7B). A total of 870, 3,097, and 2,983 genes were differentially expressed between larvae and female, larvae and pupae, and larvae and male, respectively, and 269 of these genes were common to females, males, pupae, and larvae (Fig 7C). A total of 2,437, 5,161, and 2,983 genes were differentially expressed between males and females, males and pupae, and males and larvae, respectively, and 650 of these genes were common to larvae, females, pupae, and males (Fig 7D) (S16–S21 Texts).

**Fig 8:**
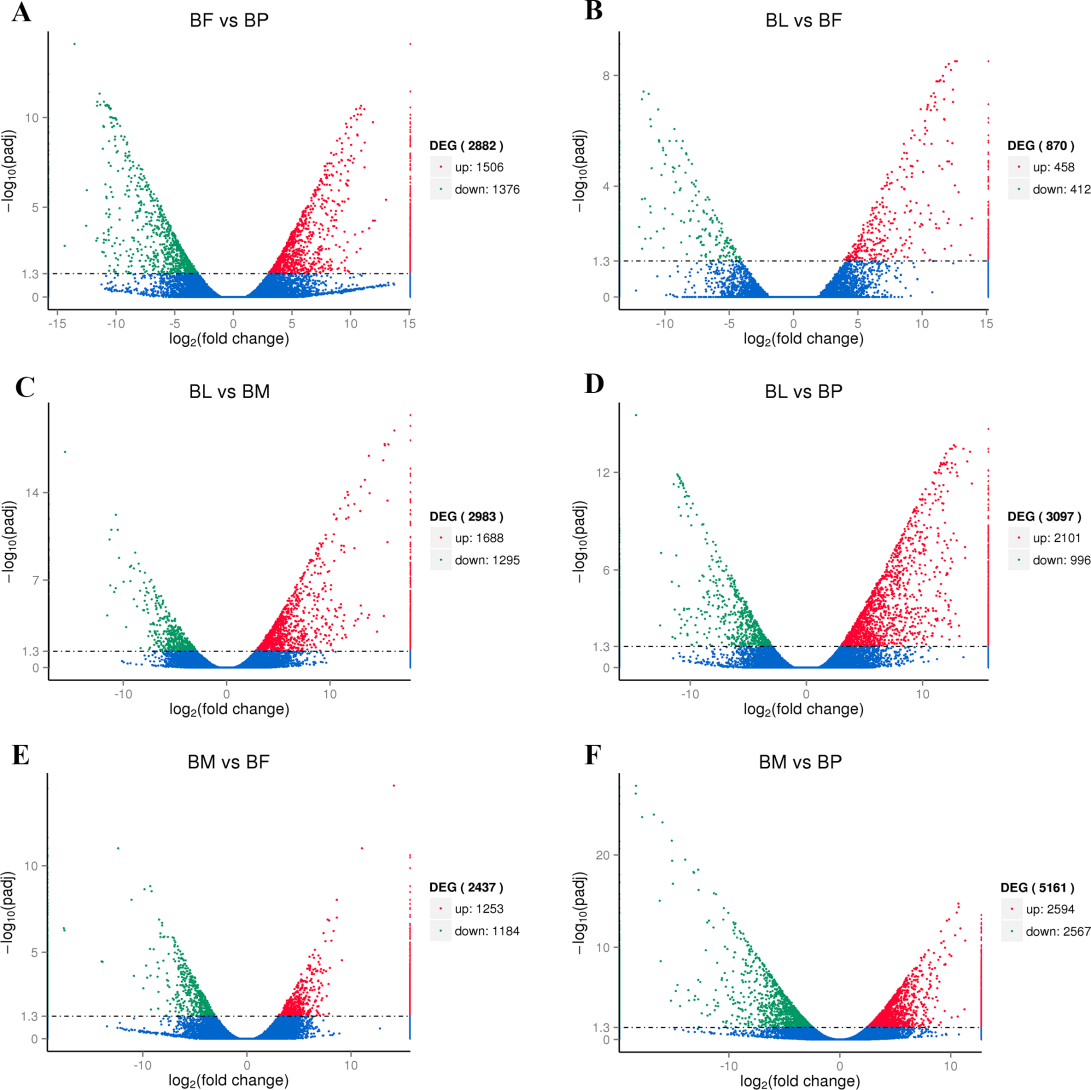
Volcano plots of differentially expressed genes in larvae, pupae, males, and females. Differentially expressed genes between (A) BF and BP, (B) BL and BF, (C) BL and BM, (D) BL and BP, (E) BM and BF and (F) BM and BP. Splashes represent different genes. Blue splashes indicate genes with no significant differential expression. Red splashes indicate significantly upregulated genes. Green splashes indicate significantly downregulated genes. BF: *B*. *horsfieldi* females, BP: *B*. *horsfieldi* pupae, BL: *B*. *horsfieldi* larvae, BM: *B*. *horsfieldi* males.

More genes were expressed in female than in pupae, in larvae than in female, in larvae than in male, in larvae than in pupae, in male than in female, and in male than in pupae (1,506, 458, 1688, 2,101, 1,253, and 2,594, respectively; Fig 8). Conversely, fewer genes were expressed in female than in pupae, in larvae than in female, in larvae than in male, in larvae than in pupae, in male than in female, and in male than in pupae (1,376, 412, 1,295, 996, 1,184, and 2,567, respectively; Fig 8).

### Phylogenetic analysis of candidate olfactory genes

**Fig 9:**
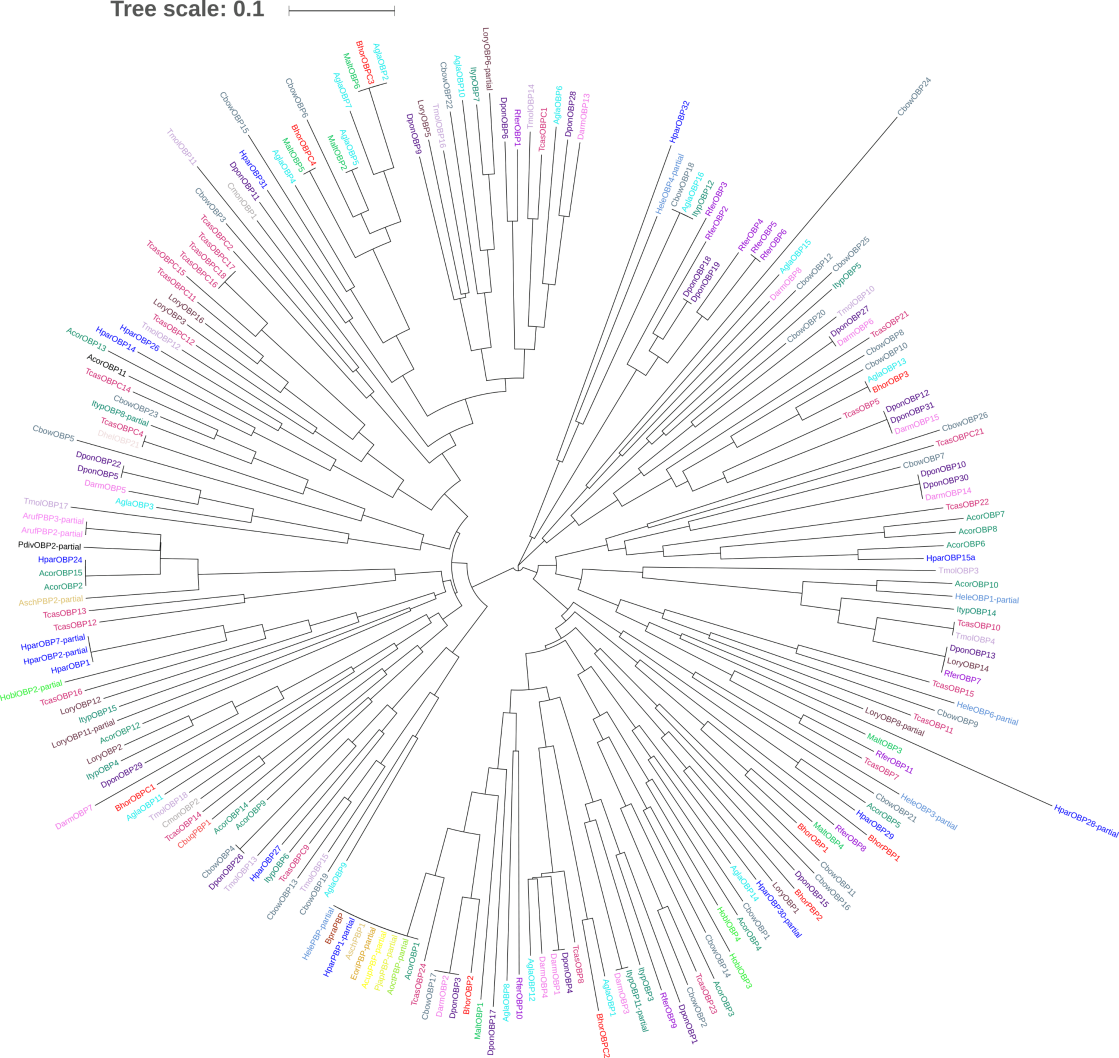
Neighbor-joining phylogenetic tree of *B*. *horsfieldi* odorant binding proteins (BhorOBPs). Values indicated at the nodes are bootstrap values based on 1000 replicates. Scale bar = 0.1. Bhor: *Batocera horsfieldi*; Tcas: *Tribolium castaneum*; Ityp: *Ips typographus*; Dpon: *Dendroctonus ponderosae*; Acor: *Anomala corpulenta*; Tmol: *Tenebrio molitor*; Malt: *Monochamus alternatus*; Pdiv: *Phyllopertha diversa*; Cmon: *Cryptolaemus montrouzieri*; Dhel: *Dasrarcus helophoroides*; Rfer: *Rhynchophorus ferrugineus*; Cbow: *Colaphellus bowringi*; Lory: *Lissorhoptrus oryzophilus*; Hobl: *Holotrichia oblita*; Agla: *Anoplophora glabripennis*; Hele: *Hylamorpha elegans*; Darm: *Dendroctonus armandi*; Hpar: *Holotrichia parallela*; Bpra: *Brachysternus prasinus*; Pjap: *Popillia japonica*; Eori: *Exomala orientalis*; Acup: *Anomala cuprea*; Asch: *Anomala schonfeldti*; Aoct: *Anomala octiescostata*; Aruf: *Anomala rufocuprea*; Cbuq: *Cyrtotrachelus buqueti*. The olfactory genes from different species are marked with different colors (S24 Text).

**Fig 10:**
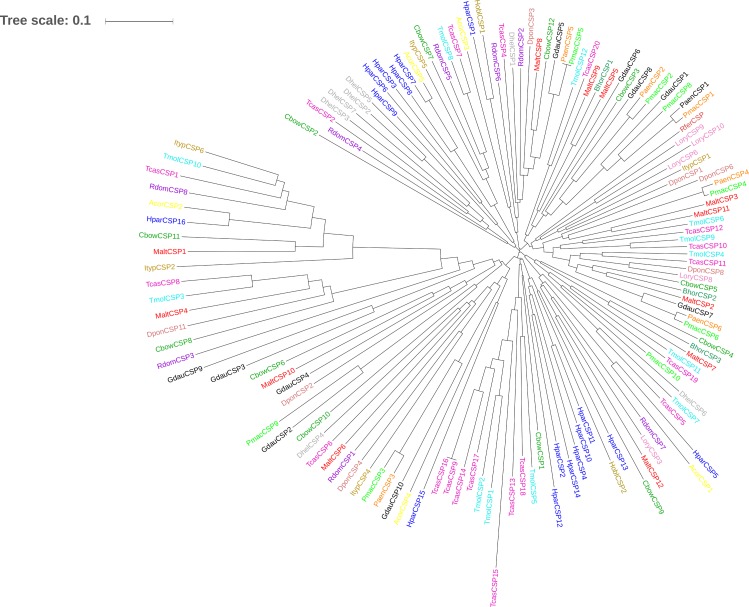
Neighbor-joining phylogenetic tree of *B*. *horsfieldi* chemosensory proteins (BhorCSPs). Values indicated at the nodes are bootstrap values based on 1000 replicates. Scale bar = 0.1. Hpar: *Holotrichia parallela*; Tmol: *Tenebrio molitor*; Ityp: *Ips typographus*; Dpon: *Dendroctonus ponderosae*; Tcas: *Tribolium castaneum*; Acor: *Anomala corpulenta*; Bhor: *Batocera horsfieldi*; Gdau: *Galeruca daurica*; Paen: *Pyrrhalta aenescens*; Pmac: *Pyrrhalta maculicollis*; Dhel: *Dasrarcus helophoroides*; Cbow: *Colaphellus bowringi*; Malt: *Monochamus alternatus*; Rdom: *Rhyzopertha dominica*; Hobl: *Holotrichia oblita*; Lory: *Lissorhoptrus oryzophilus*; Rfer: *Rhynchophorus ferrugineus*. The olfactory genes from different species are marked with different colors (S25 Text).

We constructed two phylogenetic trees comparing *BhorOBP1/2/3*, *BhorOBPC1/2/3/4* (minus-C OBP1/2/3/4) and the OBPs from 25 coleopteran insects, and *BhorCSP1/2/3* and the CSPs from 16 coleopteran insects, respectively (Figs 9 and 10). Four minus-C OBPs from *B*. *horsfieldi* grouped together with the OBPs of other coleopteran species, whereas three OBPs of *B*. *horsfieldi* separated into different clades (Fig 9). The candidate CSPs, *BhorCSP1*, *BhorCSP2*, *and BhorCSP3*, separated into different clades (Fig 10).

### Expression profiles of candidate olfactory genes

We identified 10 candidate CSPs and 16 candidate OBPs by searches against the Nr database (Deduced amino acid sequences are listed in S22 Text). Significant differences were detected in the expression profiles of the 10 candidate CSPs and 16 candidate OBPs in male and female adults (Tables 3 and 4, respectively).

**Table 3:** Differentially expressed CSP between males and females.

Gene	Readcount-Male	Readcount-Female	log2Fold-change	q
Cluster-8309.12499	144.0953	7.6845	4.2280	<0.005*
Cluster-8309.21386	283.1867	103.2319	1.4569	>0.005
Cluster-8309.22628	320.1960	200.2058	0.6775	>0.005
Cluster-8309.36051	1,276.0900	660.3976	0.9503	>0.005
Cluster-8309.36377	5,298.4300	6,861.21	-0.3729	>0.005
Cluster-8309.36573	7,629.0900	8,266.90	-0.1158	>0.005
Cluster-8309.41714	439.4133	44.2061	3.3133	<0.005*
Cluster-8309.45810	527.3126	112.3486	2.2307	>0.005
Cluster-8309.52580	1,218.3300	186.9083	2.7045	>0.005
Cluster-8309.54261	545.3564	63.9106	3.0931	<0.005*

Q values were calculated according to the method of Anders et al., 2003.

*q < 0.005 is significantly different.

**Table 4:** Differentially expressed OBPs between males and females.

Gene	Readcount-Male	Readcount-Female	log2Fold-change	q
Cluster-8309.12425	174.0043	102.9302	0.7575	>0.005
Cluster-8309.20864	122.1861	840.4307	-2.8192	<0.005*
Cluster-8309.27359	5,568.7170	720.3645	2.8177	<0.005*
Cluster-8309.31830	8,752.9100	1,295.3270	2.7564	<0.005*
Cluster-8309.32140	186.4977	61.9745	1.5894	>0.005
Cluster-8309.36213	2,129.661	44,723.7300	-4.3923	<0.005*
Cluster-8309.36426	57,197.9800	32,936.1900	0.7963	>0.005
Cluster-8309.37524	890.3307	208.1874	2.0965	>0.005
Cluster-8309.38445	5,044.1540	10,089.0100	-1.0001	>0.005
Cluster-8309.39085	217.9598	163.1848	0.4176	>0.005
Cluster-8309.39777	682.6873	1416.6270	-1.0532	>0.005
Cluster-8309.40165	399.8992	354.8279	0.1725	>0.005
Cluster-8309.40672	421.7275	641.6447	-0.6055	>0.005
Cluster-8309.41624	3,1696.7900	494.2113	6.0031	<0.005*
Cluster-8309.47478	4,136.7770	521.3067	2.7235	<0.005*
Cluster-8309.59754	589.7868	60.3018	3.0721	<0.005*

Q values were calculated according to the method of Anders et al., 2003.

*q < 0.005 is significantly different.

To further explore the olfactory genes, a local BLAST search was performed against the *B*. *horsfieldi* unigene database using the known olfactory gene sequences of *Lissorhoptrus oryzophilus*, *Monochamus alternatus*, and *Dendroctonus ponderosae* as queries. The calculated expression values based on the FKPM method of all the candidate olfactory genes are listed in Table 5.

**Table 5:** Detailed information on the candidate olfactory genes of *B*. *horsfieldi*.

**Gene name**	**Unigene ID**	**Gene length**	**Status**	**FPKM (Pupae/Larvae/Male/Female)**	**BLASTx best hit**	**Gene ID**
**BhorOBP1**	Cluster-8309.27359	1699	Complete ORF	15.50/16.36/269.25/23.25	odorant binding protein [Lissorhoptrus oryzophilus]	KC461118.1
**BhorOBP2**	Cluster-8309.40672	695	Complete ORF	30.37/311.36/66.44/59.51	odorant binding protein 6 [Monochamus alternatus]	KC461116.1
**BhorOBP3**	Cluster-8309.41624	2778	Complete ORF	18.53/233.05/872.11/7.41	odorant-binding protein 2 [Monochamus alternatus]	KC461117.1
**BhorOBP C1**	Cluster-8309.39777	739	Complete ORF	0.57/56.57/97.70/118.34	minus-C odorant binding protein 1 [Batocera horsfieldi]	GU575294.1
**BhorOBP C2**	Cluster-8309.59754	943	Complete ORF	144.19/60.64/59.26/9.97	minus-C odorant binding protein 2 [Batocera horsfieldi]	GU575295.1
**BhorOBP C3**	Cluster-8309.47478	574	Complete ORF	369.84/9.13/890.61/88.60	minus-C odorant binding protein 4 [Batocera horsfieldi]	GU584933.1
**BhorOBP C4**	Cluster-8309.31830	1229	Complete ORF	130.68/44.54/623.72/51.82	minus-C odorant binding protein 4 [Batocera horsfieldi]	GU584934.1
**BhorCSP1**	Cluster-8309.12499	888	Complete ORF	2.10/778.25/15.60/0.48	chemosensory protein [Batocera horsfieldi]	HQ587040.1
**BhorCSP2**	Cluster-8309.41714	432	Complete ORF	15.04/536.49/167.20/11.72	chemosensory protein 8 [Lissorhoptrus oryzophilus]	HQ587041.1
**BhorCSP3**	Cluster-8309.54261	2016	Complete ORF	13.50/7.44/21.38/1.38	chemosensory protein 4 [Dendroctonus ponderosae]	HQ587042.1

### Tissue- and sex-specific expressions of candidate olfactory genes

**Fig 11:**
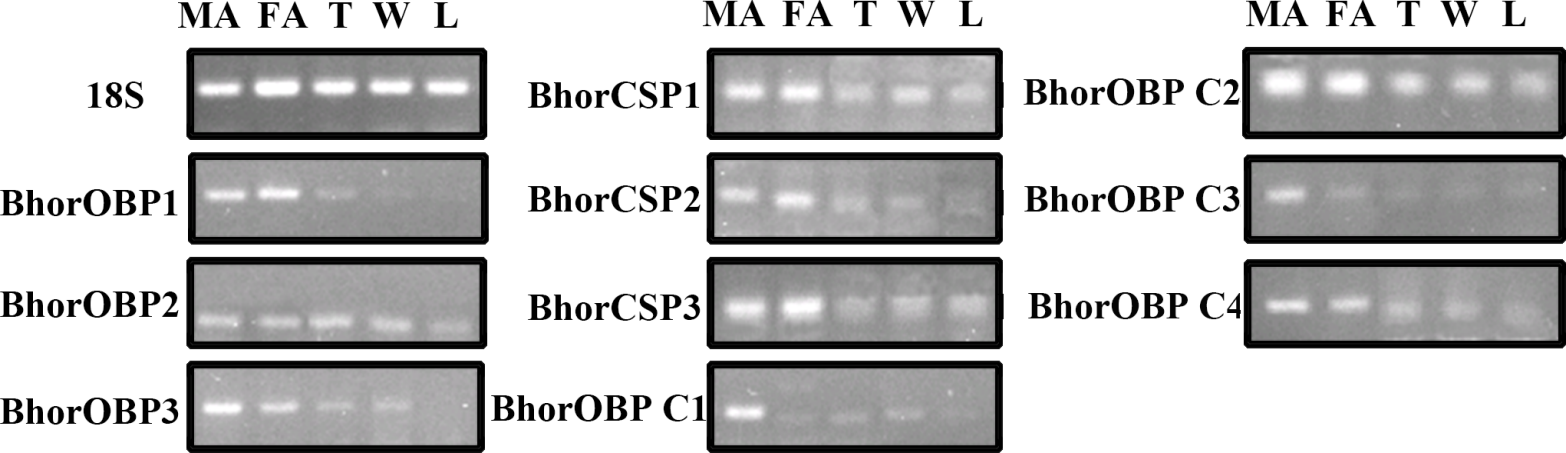
Tissue-specific expressions of candidate olfactory genes of *B*. *horsfieldi* by RT-PCR. FA: female antenna; MA: male antenna; T: thorax; W: hind wing; L: leg. 18S RNA was used as an internal control.

Semi-quantitative RT-PCR showed that all of the tested candidate olfactory genes were primarily male antenna specific (Fig 11) (S23 Text). *BhorOBP2/C2* and *BhorCSP1* showed olfactory and non-olfactory tissue expression, whereas *BhorOBP1/3*, *BhorOBPC1/C3/C4*, and *BhorCSP2/3* showed olfactory tissue-specific expression. *BhorOBP3* and *BhorOBPC1/C3* were highly expressed in the antenna of males.

**Fig 12:**
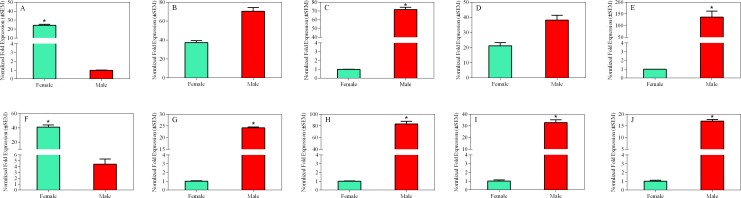
Sex-specific expressions of candidate olfactory genes of *B*. *horsfieldi* by RT-qPCR. The expression levels of male and female adults are indicated by red and green bars, respectively. Expression levels were calculated by the 2^−ΔΔCT^ method with three biological repeats. Asterisk shows a significant expression level (Chi-square test, *p*<0.05). (A–J) Sex-specific differentially expressed genes between male and female adults. (A) *BhorOBP1*, (B) *BhorOBP2*, (C) *BhorOBP3*, (D) *BhorCSP1*, (E) *BhorCSP2*, (F) *BhorCSP3*, (G) *BhorOBPC1*, (H) *BhorOBPC2*, (I) *BhorOBPC3*, (J) *BhorOBPC4*.

The RT-qPCR results were mostly consistent with the RT-PCR results; *BhorOBP2* and *BhorCSP1* showed high non-sex-specific expression, whereas *BhorOBP1/3*, *BhorCSP2/3*, and *BhorOBPC1/C2/C3/C4* showed high sex-specific expression (Fig 12).

## Discussion

Establishing insect transcription libraries for high-throughput sequencing is an important approach for molecular biology studies of insects. Transcriptome data can be used to detect new genes, transcription sites, and differentially expressed genes, as well as obtain functional gene information and transcription expression abundance, and the data can be used for molecular marker development, gene expression analysis, and small RNA analysis. [56–59]. Transcriptome for many insects has been established and analyzed, including *Ericerus pela* [60], *M*. *alternatus* [61], *Timema cristinae* [62], *Cyrtotrachelus buqueti* [53], *Drosophila melanogaster* [63], *Bombyx mori* [64], and *Bombus terrestris* [65].

In the present study, developmental transcriptomes were established for *B*. *horsfieldi* at various stages, mixed-age larvae, pupae, and male and female adults, and a relatively comprehensive gene pool was obtained. The alignments against the Nr database showed that 57.1% and 15.3% of the *B*. *horsfieldi* unigenes were similar to *T*. *castaneum* and *D*. *ponderosae* sequences, respectively. Thousands of differentially expressed genes were identified, facilitating developmental and evolutionary studies of *B*. *horsfieldi*, and contribute to future work in *B*. *horsfieldi* comparative genomics. Insects can sense changes in odorant substances in the environment through olfactory receptors, which transform chemical signals from odorant substances into electrophysiology signals that can generate various kinds of behaviors [11]. Proteins are required for odorant substances to interact with insect olfactory receptors, and for turning the chemical signals into electrophysiology signals [66]. These proteins are related to the olfactory sensation of insects and participate in the transmission of a series of signals in the insect olfactory system [67]. Moreover, olfactory proteins have been shown to act in insect nutrient uptake, life span, and behavior changes during developmental stages [67–69]. The developmental transcriptomes of *B*. *horsfieldi* provide an opportunity to understand the relationship between olfactory proteins and development. The evolution analysis of the OBPs of *B*. *horsfieldi* and 25 Coleoptera species and the CSPs of *B*. *horsfieldi* and 16 Coleoptera species showed that the OBPs of *B*. *horsfieldi* were quite similar to those of *Anoplophora glabripennis* and the CSPs of *B*. *horsfieldi* were quite similar to those of *Monochamus alternates*. Therefore, we speculated that these genes may have evolved from the same ancestral gene, but differentiated by adaptation to different types of environmental chemical factors during evolution, and perform the same or similar functions among different species [53].

Seven candidate OBPs and three candidate CSPs in male and female adults, showed significant differences in expression. To further explore the significant differences among these genes, a local BLAST was performed on the *B*. *horsfieldi* unigene database based on the known olfactory sequences of *L*. *oryzophilus*, *M*. *alternatus*, *D*. *ponderosae*, and *B*. *horsfieldi*. *BhorOBP1/2/3/C1/C2/C3/C4* were annotated as encoding odorant binding protein domains, while *BhorCSP1/2/3* were annotated as encoding chemosensory protein domains. The cloning and functional analysis of olfactory genes will be the focus of the next research.

Combining RT-PCR and RT-qPCR data, most of the candidate olfactory genes were shown to have male-specific expression patterns in *B*. *horsfieldi*, suggesting that the olfactory system is highly developed in male and that olfactory detection plays a relatively important role in males. This result supports the existence of a contact sex pheromone that is produced by *B*. *horsfieldi* female, as previously shown on the molecular level [70]. Additionally, components of the female-produced sex pheromone have been identified in other longhorn beetles, such as *Prionus californicus* [71], *Migdolus fryanus* [72], *Vesperus xatarti* [73], and *Ortholeptura valida* [74].

*BhorOBP1/3*, *BhorOBPC1/C3/C4*, and *BhorCSP2/3* showed olfactory-specific expression, suggesting that these candidate olfactory genes may play key roles in foraging and host-orientation in *B*. *horsfieldi*. A comprehensive, good-quality sequence resource from the developmental transcriptomes of *B*. *horsfieldi* larvae, pupae, female and male adults was constructed in this study. This resource enriches what is known about *B*. *horsfieldi* genomics, thus facilitating our understanding of metamorphosis, development, and fitness to environmental change. Several potential functional olfactory genes were identified. Future studies aimed at exploring the functions of these genes are the next logical step.

## Supporting information

S1 Text Female1 annotation. (DOCX)

S2 Text Female2 annotation. (XLS)

S3 Text Female3 annotation. (XLS)

S4 Text Larvae1 annotation. (XLS)

S5 Text Larvae2 annotation. (XLS)

S6 Text Larvae3 annotation. (XLS)

S7 Text Male1 annotation. (XLS)

S8 Text Male2 annotation. (XLS)

S9 Text Male3 annotation. (XLS)

S10 Text Pupae1 annotation. (XLS)

S11 Text Pupae2 annotation. (XLS)

S12 Text Pupae3 annotation. (XLS)

S13 Text Annotation GO. (XLS)

S14 Text Annotation KEGG. (XLS)

S15 Text Annotation KOG. (XLS)

S16 Text BF vs BP. DEG. (XLS)

S17 Text BL vs BF. DEG. (XLS)

S18 Text BL vs BM. DEG. (XLS)

S19 Text BL vs BP. DEG. (XLS)

S20 Text BM vs BF. DEG. (XLS)

S21 Text BM vs BP. DEG. (XLS)

S22 Text Deduced amino acid sequences of candidate olfactory genes. (DOCX)

S23 Text The genbank number and open reading frame of candidate olfactory genes. (DOCX)

S24 Text The dataset and accession number (OBPs). (DOCX)

S25 Text The dataset and accession number (CSPs). (DOCX)

S1 Table Transcriptome software and parameters list. (XLS)

S2 Table Nucleotide sequences of candidate olfactory genes. (DOCX)

S3 Table Primer used in RT-PCR and RT-qPCR. (DOCX)
