# Correction: Transcriptome analysis in different developmental stages of *Batocera horsfieldi* (Coleoptera: Cerambycidae) and comparison of candidate olfactory genes

**DOI:** 10.1371/journal.pone.0214472

**Published:** 2019-03-21

**Authors:** Hua Yang, Yan Cai, Zhihang Zhuo, Wei Yang, Chunping Yang, Jin Zhang, Yang Yang, Baoxin Wang, Fengrong Guan

The Data Availability statement for this paper is incorrect. The correct statement is: Data underlying this manuscript is available on the NCBI SRA database under BioProject number PRJNA508447 (https://www.ncbi.nlm.nih.gov/sra/?term=PRJNA508447).
