# Supplementary material for: Transcriptome analysis in different developmental stages of Batocera horsfieldi (Coleoptera: Cerambycidae) and comparison of candidate olfactory genes
Source: PLoS One. 2018 Feb 23;13(2):e0192730. doi: 10.1371/journal.pone.0192730 (PMC5825065; doi:10.1371/journal.pone.0192730)
Supplement: S1 Text — (DOCX) [file pone.0192730.s001.docx]

**Table S1** Transcriptome software and parameters list

| Software | Version | Parameters | Remark |
| --- | --- | --- | --- |
| Trinity | r20140413p1 | min_kmer_cov: 2 | - |
| NCBI blast 2.2.28+ | v2.2.28+ | NR, NT, Swiss-Prot: e-value = 1.0e-5; KOG/COG: e-value = 1.0e-3 | Nr, Nt, KOG/COG, Swiss-Prot |
| KAAS | r140224 | e-value = 1.0e-10 | KEGG |
| hmmscan | HMMER 3 | e-value = 0.01 | Pfam |
| blast2go | b2g4pipe_v2.5 | e-value = 1.0e-6 | GO |
| RSEM | v1.2.15 | bowtie2 mismatch0 | - |
| GATK2 | v3.2 | QUAL<30.0 QD<5.0 | SNP |
| MISA, primer3 | primer3-2.3.4 | SSR: 1-10 2-6 3-5 4-5 5-5 6-5 | SSR |
| DEGSeq | 1.12.0 | padj&lt0.05 | - |
| DESeq | 1.10.1 |  | - |
| edgeR | 3.0.8 |  | - |
| GOSeq, topGO | 1.10.0, 2.10.0 | Corrected P-Value&lt0.05 | - |
| KOBAS | v2.0.12 | Corrected P-Value&lt0.05 | - |
| NCBI blast 2.2.28+ | v2.2.28+ | e-value = 1.0e-10 | Analysis of protein interactions |
