# Supplementary material for: Transcriptome analysis in different developmental stages of Batocera horsfieldi (Coleoptera: Cerambycidae) and comparison of candidate olfactory genes
Source: PLoS One. 2018 Feb 23;13(2):e0192730. doi: 10.1371/journal.pone.0192730 (PMC5825065; doi:10.1371/journal.pone.0192730)
Supplement: S22 Text — (DOCX) [file pone.0192730.s022.docx]

>BhorOBP1

MKLFVFVLCLCLATGCAYSAFTEKQLNATKKLMRNTCQNKAKPTSEQIDAMHRGEFSDDRNAQCYLLCIMNTYKLLTKENTFDWESGVKALAANAPPSIGDPGIVSIKNCKDAVKTTSDKCVASTEIAKCIYNDNPSNYFLP

>BhorOBP2

MSLRIVIALFSLASVAYAKLQLPVELQEYADGLHDLCIKKTGITEDDHIAYDIANNPHDEKLQCYIKCLMLEANWMDKDGTIQYAWIEENLHQDVKDIVITALSKCKNINEGANLCEKASHFNACMYEADKENWFLV

>BhorOBP3

MVASMSIVSLVISILAVHAQFDKLPDGKIPPEILKCSESVGLQPKGKPMLTREPSSEEMCFFKCIMEEKGMLDADGNVKPETVDSSQLHIPQDKVDDVKQCLKNAGKVEKCEDIAKLVECMPQPA

>BhorCSP1

MKLVVLLLFVALCGMAYGRPDDGKYTTKYDNIDLDEILKNDRLLRAYVDCLKGTKKCTNDGEELKKVLPEAIDNDCAKCNDTQKNGARKVIRYLIKNKRDWWNELEVIYDPTGKYKKKYEEEAKKEGLEV

>BhorCSP2

MKAYLSFVALLVAVACARADDDKYTTKYDNVDLDEIVKSDRLLKNYVNCLLEKGNCTPDGTELKKVLPDALLTDCTKCSDTQKKGSKKIIRHLIDNKADWYKELEAKYDKDGVYKKKYEEELELKKE

>BhorCSP3

MDALYELVYLALLFSSVVAEETYTTKFDNIDYEEILRSDRLLRNYINCLLDRGGCTAEGKELRRILPDALETDCSKCSETQRKAAKKVIQHLVNNKADMWEELMVMYDPDGEFKKKYEGEWLNED

>BhorOBP C1

MKTVFVVSLLFALAASDTDMEKKFHECDEETGLTLSEVTEYLLGDDAENDEKATKYMMCMFKQQGAIDGEGHLDMEKVRLSVNNYMKTTDAADDKEALECVEEKDTAEETALAVGKCVEKRRAELTSSK

>BhorOBP C2

MDSLIFLVVVSSLLAMSTVQAALERSEYSPKLLELVDSLHSICIGKSGTDEDSINKVINGEFTDEPKIKKYMKCGITEVGVMNEEGVIDYEMTAELLPVKLVDKSIAIIKKCEADGKDIPNLDDRVFALFKCYHDQDPETFIFF

>BhorOBP C3

MKIFLVLLCTIVGIWAQENKKLIAEEQMLEHIHDECQADPATNADHELLHNLAANIDNPQVGAHMLCESTKVGLQKPNGELDIPTIKEKIGLSVPDANRVEFLVKECAIKKNTPEKTAINLFMCLDKNGVTYFHEF

>BhorOBP C4

MKTAFVFACVVVAALAASLSEEEKKLQEIHDKCQADPATYVDHELLHNLSANIDNPKVGAHMLCESKAVGLQKPNGELDLNVIKQKISLTVSDKAKVERLVKECAVKKQTPEKTAVNLFMCLDKDGVTYFHEF
