# Supplementary material for: Transcriptome analysis in different developmental stages of Batocera horsfieldi (Coleoptera: Cerambycidae) and comparison of candidate olfactory genes
Source: PLoS One. 2018 Feb 23;13(2):e0192730. doi: 10.1371/journal.pone.0192730 (PMC5825065; doi:10.1371/journal.pone.0192730)
Supplement: S23 Text — (DOCX) [file pone.0192730.s023.docx]

NO: KC461118.1 BhorOBP1

ATGAAACTTTTTGTATTCGTCCTGTGCTTATGTCTTGCCACAGGTTGCGCGTACTCAGCGTTTACAGAAAAGCAGCTAAACGCTACCAAAAAACTTATGCGAAATACGTGTCAAAATAAGGCTAAACCAACGAGTGAGCAAATAGATGCCATGCATAGAGGTGAATTTAGTGATGACAGAAACGCCCAGTGTTACCTTCTTTGTATAATGAACACATACAAATTGTTAACCAAAGAAAATACATTCGATTGGGAAAGCGGTGTTAAAGCACTTGCTGCCAATGCTCCACCTAGTATTGGCGACCCGGGCATTGTCAGTATCAAAAATTGCAAAGATGCCGTTAAAACGACCAGTGACAAATGTGTAGCTTCCACAGAAATTGCCAAGTGTATATATAATGACAATCCCTCGAATTATTTCTTGCCCTAA

NO: KC461116.1 BhorOBP2

ATGTCGCTCAGAATCGTTATCGCCCTTTTCAGTTTAGCTAGCGTGGCTTACGCCAAATTGCAACTTCCAGTTGAATTACAGGAATATGCAGATGGTTTGCACGATTTGTGTATTAAGAAAACGGGAATTACAGAGGACGACCACATAGCATATGATATAGCAAACAATCCACACGACGAAAAACTTCAATGCTATATAAAGTGCCTTATGTTAGAGGCAAATTGGATGGATAAAGATGGCACTATACAGTACGCCTGGATTGAGGAGAACCTACATCAAGACGTTAAGGATATAGTAATCACAGCCCTTAGTAAATGCAAAAATATAAACGAAGGTGCAAATCTTTGCGAAAAAGCTTCACACTTCAACGCCTGCATGTACGAAGCTGATAAGGAGAACTGGTTCCTGGTATAG

NO: KC461117.1 BhorOBP3

ATGGTCGCTTCAATGAGTATTGTTAGTTTGGTGATATCTATCTTGGCGGTTCATGCACAATTCGACAAGTTGCCAGATGGTAAGATACCGCCCGAAATTCTAAAATGCTCAGAGTCTGTGGGACTTCAACCAAAAGGTAAACCAATGTTAACGAGAGAACCCAGTTCCGAGGAGATGTGTTTCTTCAAATGCATCATGGAGGAGAAAGGCATGCTGGACGCAGACGGAAATGTAAAACCGGAAACTGTGGACAGTTCACAGCTCCATATCCCGCAGGATAAAGTGGACGACGTCAAACAATGCTTGAAGAATGCTGGCAAGGTCGAGAAGTGCGAGGATATAGCCAAGTTGGTAGAATGCATGCCGCAGCCCGCTTGA

NO: HQ587040.1 BhorCSP1

ATGAAATTAGTCGTGCTCTTACTTTTCGTGGCCCTCTGCGGCATGGCCTACGGACGTCCTGACGACGGCAAATATACCACCAAATACGACAACATCGATCTGGACGAGATCCTGAAGAACGACAGGCTGCTGAGGGCGTACGTGGACTGTCTTAAGGGCACCAAAAAGTGCACTAATGACGGCGAAGAGCTGAAAAAGGTACTACCCGAAGCTATAGACAACGATTGTGCCAAATGCAACGACACCCAGAAGAACGGTGCCAGAAAAGTGATCCGCTACCTCATCAAGAACAAGCGCGACTGGTGGAACGAACTGGAGGTCATCTACGACCCAACCGGTAAATACAAGAAGAAATACGAAGAAGAAGCCAAGAAGGAGGGTCTCGAAGTATAA

NO: HQ587041.1 BhorCSP2

ATGAAGGCCTATCTGTCGTTTGTCGCACTCCTCGTCGCGGTGGCGTGTGCACGTGCCGACGACGACAAGTACACCACCAAATACGACAACGTGGACCTCGACGAGATCGTCAAGAGCGACCGCCTGCTGAAGAACTACGTAAACTGCTTGCTGGAAAAGGGAAACTGTACTCCCGATGGAACTGAACTGAAGAAGGTTCTTCCTGACGCCCTCCTCACCGACTGTACGAAATGCAGCGACACCCAGAAGAAAGGGAGCAAGAAAATCATCCGCCACTTGATCGACAACAAGGCCGATTGGTACAAGGAACTCGAGGCTAAATACGACAAAGACGGCGTATACAAGAAGAAATACGAAGAAGAGTTAGAGCTTAAGAAAGAATAA

NO: HQ587042.1 BhorCSP3

ATGGATGCGTTATACGAACTAGTGTATTTGGCGTTACTATTCTCTTCGGTCGTTGCAGAAGAAACGTACACCACAAAGTTTGACAATATAGACTATGAGGAAATCCTAAGAAGCGATCGTCTTTTGAGAAATTATATAAATTGTCTTTTAGACAGGGGAGGTTGCACTGCTGAGGGAAAAGAACTAAGGAGAATCTTGCCGGATGCTTTGGAAACGGATTGTTCCAAGTGCAGCGAAACTCAACGAAAGGCGGCAAAGAAAGTCATACAGCATTTGGTGAACAACAAGGCGGACATGTGGGAGGAACTGATGGTCATGTACGATCCCGATGGGGAGTTTAAGAAGAAATACGAGGGCGAATGGCTAAATGAGGACTAA

NO: GU575294.1 BhorOBP C1

ATGAAAACTGTGTTTGTGGTGTCTCTCTTGTTCGCACTAGCGGCTTCAGATACCGATATGGAGAAGAAATTTCACGAATGTGATGAAGAAACGGGCTTAACATTATCAGAAGTAACAGAATATCTTCTTGGAGACGACGCTGAGAACGACGAAAAAGCGACTAAATATATGATGTGTATGTTCAAACAACAAGGAGCCATCGACGGCGAAGGCCATCTCGATATGGAGAAGGTCCGACTGTCGGTAAACAACTACATGAAGACGACAGATGCCGCCGACGACAAAGAGGCTTTGGAGTGCGTCGAGGAGAAGGACACGGCCGAAGAAACGGCCCTCGCAGTCGGCAAGTGCGTGGAGAAAAGAAGAGCTGAACTTACTAGTTCAAAATAA

NO: GU575295.1 BhorOBP C2

ATGGATAGCTTAATATTTCTAGTAGTGGTGTCTTCGCTCTTAGCGATGTCTACCGTCCAAGCCGCCTTGGAGCGATCTGAATATAGTCCGAAACTTCTGGAATTAGTGGACTCGCTGCATTCGATTTGTATAGGAAAGTCAGGAACGGATGAAGATTCCATTAATAAAGTCATAAACGGAGAATTTACCGATGAACCTAAAATTAAGAAATATATGAAGTGCGGCATTACGGAAGTTGGAGTGATGAATGAGGAAGGCGTTATCGACTATGAGATGACAGCTGAACTGCTTCCGGTGAAACTAGTGGATAAAAGTATAGCAATTATAAAAAAATGCGAAGCAGATGGAAAAGATATTCCAAACCTTGACGATAGAGTATTCGCTTTATTTAAATGCTATCATGACCAAGATCCAGAAACATTTATTTTCTTCTAA

NO: GU584933.1 BhorOBP C3

ATGAAAATCTTTCTTGTGTTATTGTGTACTATCGTCGGCATCTGGGCGCAAGAGAATAAAAAGCTCATCGCCGAAGAGCAGATGTTGGAGCACATCCACGACGAGTGTCAGGCCGACCCCGCCACCAACGCCGACCATGAATTACTCCACAACCTCGCCGCAAACATCGACAACCCACAAGTAGGCGCCCACATGCTCTGCGAGTCCACCAAGGTGGGACTTCAGAAGCCAAACGGAGAATTGGACATCCCGACCATCAAAGAGAAGATCGGCCTCTCCGTCCCAGATGCAAATAGAGTCGAATTTTTGGTGAAGGAATGCGCTATTAAGAAGAACACCCCGGAGAAGACCGCCATCAACCTATTCATGTGTTTGGACAAAAACGGTGTTACCTACTTCCATGAGTTTTAA

NO: GU584934.1 BhorOBP C4

ATGAAAACTGCCTTTGTCTTCGCTTGCGTCGTCGTCGCAGCTTTGGCTGCCAGCCTTTCCGAAGAGGAGAAGAAACTCCAGGAAATCCACGACAAATGCCAAGCCGACCCAGCCACCTACGTCGACCACGAATTGTTGCACAACCTCTCCGCAAACATCGACAACCCCAAAGTTGGCGCCCACATGCTCTGCGAATCCAAAGCCGTCGGTCTCCAGAAGCCAAACGGCGAATTGGACTTGAATGTCATCAAGCAGAAGATCTCCCTCACCGTCAGCGACAAGGCCAAAGTTGAGAGGTTGGTCAAAGAATGCGCTGTGAAGAAGCAAACTCCAGAGAAGACTGCCGTCAACCTATTCATGTGCTTGGACAAGGATGGAGTTACTTACTTCCATGAATTCTAA
