# Supplementary material for: Transcriptome analysis in different developmental stages of Batocera horsfieldi (Coleoptera: Cerambycidae) and comparison of candidate olfactory genes
Source: PLoS One. 2018 Feb 23;13(2):e0192730. doi: 10.1371/journal.pone.0192730 (PMC5825065; doi:10.1371/journal.pone.0192730)
Supplement: S24 Text — (DOCX) [file pone.0192730.s024.docx]

TcasOBP5 [*Tribolium castaneum*]. GenBank accession number EFA05677.1

TcasOBP7 [*Tribolium castaneum*]. GenBank accession number EFA04593.1

TcasOBP8 [*Tribolium castaneum*]. GenBank accession number EFA04687.2

TcasOBP10 [*Tribolium castaneum*]. GenBank accession number EFA07542.1

TcasOBP11 [*Tribolium castaneum*]. GenBank accession number EFA05695.1

TcasOBP12 [*Tribolium castaneum*]. GenBank accession number EFA02857.1

TcasOBP13 [*Tribolium castaneum*]. GenBank accession number EFA02858.1

TcasOBP14 [*Tribolium castaneum*]. GenBank accession number EFA02914.1

TcasOBP15 [*Tribolium castaneum*]. GenBank accession number EFA12066.1

TcasOBP16 [*Tribolium castaneum*]. GenBank accession number EFA02853.2

TcasOBP21 [*Tribolium castaneum*]. GenBank accession number EFA09215.2

TcasOBP22 [*Tribolium castaneum*]. GenBank accession number EFA09155.2

TcasOBP23 [*Tribolium castaneum*]. GenBank accession number EFA10803.1

TcasOBP24 [*Tribolium castaneum*]. GenBank accession number EFA04576.1

ItypOBP3 [*Ips typographus*]. GenBank accession number JAA74394.1

ItypOBP4 [*Ips typographus*]. GenBank accession number JAA74393.1

ItypOBP5 [*Ips typographus*]. GenBank accession number JAA74392.1

ItypOBP6 [*Ips typographus*]. GenBank accession number JAA74391.1

ItypOBP7 [*Ips typographus*]. GenBank accession number JAA74390.1

ItypOBP8-partial [*Ips typographus*]. GenBank accession number JAA74389.1

ItypOBP11-partial [*Ips typographus*]. GenBank accession number JAA74401.1

ItypOBP12 [*Ips typographus*]. GenBank accession number JAA74400.1

ItypOBP14 [*Ips typographus*]. GenBank accession number JAA74398.1

ItypOBP15 [*Ips typographus*]. GenBank accession number JAA74397.1

DponOBP1 [*Dendroctonus ponderosae*]. GenBank accession number AKK25129.1

DponOBP3 [*Dendroctonus ponderosae*]. GenBank accession number AKK25131.1

DponOBP4 [*Dendroctonus ponderosae*]. GenBank accession number AKK25132.1

DponOBP5 [*Dendroctonus ponderosae*]. GenBank accession number AKK25133.1

DponOBP6 [*Dendroctonus ponderosae*]. GenBank accession number AKK25134.1

DponOBP9 [*Dendroctonus ponderosae*]. GenBank accession number AGI05185.1

DponOBP10 [*Dendroctonus ponderosae*]. GenBank accession number AKK25136.1

DponOBP11 [*Dendroctonus ponderosae*]. GenBank accession number AGI05181.1

DponOBP12 [*Dendroctonus ponderosae*]. GenBank accession number AKK25137.1

DponOBP13 [*Dendroctonus ponderosae*]. GenBank accession number AKK25138.1

DponOBP15 [*Dendroctonus ponderosae*]. GenBank accession number AKK25139.1

DponOBP17 [*Dendroctonus ponderosae*]. GenBank accession number AKK25141.1

DponOBP18 [*Dendroctonus ponderosae*]. GenBank accession number AKK25142.1

DponOBP19 [*Dendroctonus ponderosae*]. GenBank accession number AGI05183.1

DponOBP22 [*Dendroctonus ponderosae*]. GenBank accession number AGI05180.1

DponOBP26 [*Dendroctonus ponderosae*]. GenBank accession number AGI05183.1

DponOBP27 [*Dendroctonus ponderosae*]. GenBank accession number AGI05187.1

DponOBP28 [*Dendroctonus ponderosae*]. GenBank accession number AGI05178.1

DponOBP29 [*Dendroctonus ponderosae*]. GenBank accession number AGI05182.1

DponOBP30 [*Dendroctonus ponderosae*]. GenBank accession number AGI05176.1

DponOBP31 [*Dendroctonus ponderosae*]. GenBank accession number AGI05165.1

AcorOBP1 [*Anomala corpulenta*]. GenBank accession number KM251641.1

AcorOBP2 [*Anomala corpulenta*]. GenBank accession number KJ093447.1

AcorOBP3 [*Anomala corpulenta*]. GenBank accession number KM251643.1

AcorOBP4 [*Anomala corpulenta*]. GenBank accession number KM251644.1

AcorOBP5 [*Anomala corpulenta*]. GenBank accession number AKC58526.1

AcorOBP6 [*Anomala corpulenta*]. GenBank accession number KM251646.1

AcorOBP7 [*Anomala corpulenta*]. GenBank accession number KM251647.1

AcorOBP8 [*Anomala corpulenta*]. GenBank accession number KM251648.1

AcorOBP9 [*Anomala corpulenta*]. GenBank accession number AKC58530.1

AcorOBP10 [*Anomala corpulenta*]. GenBank accession number AKC58531.1

AcorOBP11 [*Anomala corpulenta*]. GenBank accession number KM251651.1

AcorOBP12 [*Anomala corpulenta*]. GenBank accession number KM251652.1

AcorOBP13 [*Anomala corpulenta*]. GenBank accession number KM251653.1

AcorOBP14 [*Anomala corpulenta*]. GenBank accession number AKC58520.1

AcorOBP15 [*Anomala corpulenta*]. GenBank accession number AKC58521.1

TmolOBP3 [*Tenebrio molitor*]. GenBank accession number AJM71477.1

TmolOBP4 [*Tenebrio molitor*]. GenBank accession number AJM71478.1

TmolOBP10 [*Tenebrio molitor*]. GenBank accession number AJM71484.1

TmolOBP11 [*Tenebrio molitor*]. GenBank accession number AJM71485.1

TmolOBP12 [*Tenebrio molitor*]. GenBank accession number AJM71486.1

TmolOBP13 [*Tenebrio molitor*]. GenBank accession number AJM71487.1

TmolOBP14 [*Tenebrio molitor*]. GenBank accession number AJM71488.1

TmolOBP15 [*Tenebrio molitor*]. GenBank accession number AJM71489.1

TmolOBP16 [*Tenebrio molitor*]. GenBank accession number AJM71490.1

TmolOBP17 [*Tenebrio molitor*]. GenBank accession number AJM71491.1

TmolOBP18 [*Tenebrio molitor*]. GenBank accession number AJM71492.1

MaltOBP1 [*Monochamus alternatus*]. GenBank accession number EF593044.1

MaltOBP2 [*Monochamus alternatus*]. GenBank accession number AJO67867.1

MaltOBP3 [*Monochamus alternatus*]. GenBank accession number AHA39268.1

MaltOBP4 [*Monochamus alternatus*]. GenBank accession number AHA39269.1

MaltOBP5 [*Monochamus alternatus*]. GenBank accession number AIX97020.1

MaltOBP6 [*Monochamus alternatus*]. GenBank accession number KP120892.1

PdivOBP2-partial [*Phyllopertha diverse*]. GenBank accession number AB026553.1

CmonOBP1 [*Cryptolaemus montrouzieri*]. GenBank accession number KU170686.1

CmonOBP2 [*Cryptolaemus montrouzieri*]. GenBank accession number KU170685.1

DhelOBP21 [*Dastarcus helophoroides*]. GenBank accession number KF984184.1

RferOBP1 [*Rhynchophorus ferrugineus*]. GenBank accession number KR780571.1

RferOBP2 [*Rhynchophorus ferrugineus*]. GenBank accession number KR780572.1

RferOBP3 [*Rhynchophorus ferrugineus*]. GenBank accession number KR780573.1

RferOBP4 [*Rhynchophorus ferrugineus*]. GenBank accession number KR780574.1

RferOBP5 [*Rhynchophorus ferrugineus*]. GenBank accession number KR780575.1

RferOBP6 [*Rhynchophorus ferrugineus*]. GenBank accession number KR780576.1

RferOBP7 [*Rhynchophorus ferrugineus*]. GenBank accession number KR780577.1

RferOBP8 [*Rhynchophorus ferrugineus*]. GenBank accession number KR780578.1

RferOBP9 [*Rhynchophorus ferrugineus*]. GenBank accession number KR780579.1

RferOBP10 [*Rhynchophorus ferrugineus*]. GenBank accession number KR780580.1

RferOBP11 [*Rhynchophorus ferrugineus*]. GenBank accession number KR780581.1

CbowOBP1 [*Colaphellus bowringi*]. GenBank accession number KT381483.1

CbowOBP2 [*Colaphellus bowringi*]. GenBank accession number KT381484.1

CbowOBP3 [*Colaphellus bowringi*]. GenBank accession number KT381485.1

CbowOBP4 [*Colaphellus bowringi*]. GenBank accession number KT381486.1

CbowOBP5 [*Colaphellus bowringi*]. GenBank accession number KT381487.1

CbowOBP6 [*Colaphellus bowringi*]. GenBank accession number KT381488.1

CbowOBP7 [*Colaphellus bowringi*]. GenBank accession number KT381489.1

CbowOBP8 [*Colaphellus bowringi*]. GenBank accession number KT381490.1

CbowOBP9 [*Colaphellus bowringi*]. GenBank accession number KT381491.1

CbowOBP10 [*Colaphellus bowringi*]. GenBank accession number KT381492.1

CbowOBP11 [*Colaphellus bowringi*]. GenBank accession number KT381493.1

CbowOBP12 [*Colaphellus bowringi*]. GenBank accession number KT381494.1

CbowOBP13 [*Colaphellus bowringi*]. GenBank accession number KT381495.1

CbowOBP14 [*Colaphellus bowringi*]. GenBank accession number KT381496.1

CbowOBP15 [*Colaphellus bowringi*]. GenBank accession number KT381497.1

CbowOBP16 [*Colaphellus bowringi*]. GenBank accession number KT381498.1

CbowOBP17 [*Colaphellus bowringi*]. GenBank accession number KT381499.1

CbowOBP18 [*Colaphellus bowringi*]. GenBank accession number KT381500.1

CbowOBP19 [*Colaphellus bowringi*]. GenBank accession number KT381501.1

CbowOBP20 [*Colaphellus bowringi*]. GenBank accession number KT381502.1

CbowOBP21 [*Colaphellus bowringi*]. GenBank accession number KT381503.1

CbowOBP22 [*Colaphellus bowringi*]. GenBank accession number KT381504.1

CbowOBP23 [*Colaphellus bowringi*]. GenBank accession number KT381505.1

CbowOBP24 [*Colaphellus bowringi*]. GenBank accession number KT381506.1

CbowOBP25 [*Colaphellus bowringi*]. GenBank accession number KT381507.1

CbowOBP26 [*Colaphellus bowringi*]. GenBank accession number KT381508.1

LoryOBP1 [*Lissorhoptrus oryzophilus*]. GenBank accession number KF383282.1

LoryOBP2 [*Lissorhoptrus oryzophilus*]. GenBank accession number KF383281.1

LoryOBP3 [*Lissorhoptrus oryzophilus*]. GenBank accession number KF383273.1

LoryOBP5 [*Lissorhoptrus oryzophilus*]. GenBank accession number KF383280.1

LoryOBP6-partial [*Lissorhoptrus oryzophilus*]. GenBank accession number KF383279.1

LoryOBP8-partial [*Lissorhoptrus oryzophilus*]. GenBank accession number KF383278.1

LoryOBP11-partial [*Lissorhoptrus oryzophilus*]. GenBank accession number KF383277.1

LoryOBP12 [*Lissorhoptrus oryzophilus*]. GenBank accession number KF383276.1

LoryOBP14 [*Lissorhoptrus oryzophilus*]. GenBank accession number KF383275.1

LoryOBP16 [*Lissorhoptrus oryzophilus*]. GenBank accession number KF383274.1

HoblOBP2-partial [*Holotrichia oblita*]. GenBank accession number GQ856257.2

HoblOBP3 [*Holotrichia oblita*]. GenBank accession number HQ688992.1

HoblOBP4 [*Holotrichia oblita*]. GenBank accession number HQ688993.1

AglaOBP1 [*Anoplophora glabripennis*]. GenBank accession number KX660670.1

AglaOBP2 [*Anoplophora glabripennis*]. GenBank accession number KX660671.1

AglaOBP3 [*Anoplophora glabripennis*]. GenBank accession number KX660672.1

AglaOBP4 [*Anoplophora glabripennis*]. GenBank accession number KX890101.1

AglaOBP5 [*Anoplophora glabripennis*]. GenBank accession number KX890102.1

AglaOBP6 [*Anoplophora glabripennis*]. GenBank accession number KX890103.1

AglaOBP7 [*Anoplophora glabripennis*]. GenBank accession number KX890104.1

AglaOBP8 [*Anoplophora glabripennis*]. GenBank accession number KX890105.1

AglaOBP9 [*Anoplophora glabripennis*]. GenBank accession number KX890106.1

AglaOBP10 [*Anoplophora glabripennis*]. GenBank accession number KX890107.1

AglaOBP11 [*Anoplophora glabripennis*]. GenBank accession number KX890108.1

AglaOBP12 [*Anoplophora glabripennis*]. GenBank accession number KX890109.1

AglaOBP13 [*Anoplophora glabripennis*]. GenBank accession number KX890110.1

AglaOBP14 [*Anoplophora glabripennis*]. GenBank accession number KX890111.1

AglaOBP15 [*Anoplophora glabripennis*]. GenBank accession number KX890112.1

AglaOBP16 [*Anoplophora glabripennis*]. GenBank accession number KX890113.1

HeleOBP1-partial [*Hylamorpha elegans*]. GenBank accession number KT861417.1

HeleOBP3-partial [*Hylamorpha elegans*]. GenBank accession number KT861418.1

HeleOBP4-partial [*Hylamorpha elegans*]. GenBank accession number KT861419.1

HeleOBP6-partial [*Hylamorpha elegans*]. GenBank accession number KT861420.1

DarmOBP1 [*Dendroctonus armandi*]. GenBank accession number KJ082104.1

DarmOBP2 [*Dendroctonus armandi*]. GenBank accession number KJ082105.1

DarmOBP3 [*Dendroctonus armandi*]. GenBank accession number KP453834.1

DarmOBP4 [*Dendroctonus armandi*]. GenBank accession number KP453835.1

DarmOBP5 [*Dendroctonus armandi*]. GenBank accession number KP453836.1

DarmOBP6 [*Dendroctonus armandi*]. GenBank accession number KP453837.1

DarmOBP7 [*Dendroctonus armandi*]. GenBank accession number KP453838.1

DarmOBP8 [*Dendroctonus armandi*]. GenBank accession number KP453839.1

DarmOBP13 [*Dendroctonus armandi*]. GenBank accession number KP453840.1

DarmOBP14 [*Dendroctonus armandi*]. GenBank accession number KP453841.1

DarmOBP15 [*Dendroctonus armandi*]. GenBank accession number KP453842.1

HparOBP1 [*Holotrichia parallela*]. GenBank accession number JF422903.1

HparOBP2-partial [*Holotrichia parallela*]. GenBank accession number AB026556.1

HparOBP7-partial [*Holotrichia parallela*]. GenBank accession number KR733553.1

HparOBP14 [*Holotrichia parallela*]. GenBank accession number KR733560.1

HparOBP15a [*Holotrichia parallela*]. GenBank accession number KT279201.1

HparOBP24 [*Holotrichia parallela*]. GenBank accession number KR733570.1

HparOBP26 [*Holotrichia parallela*]. GenBank accession number KT207379.1

HparOBP27 [*Holotrichia parallela*]. GenBank accession number KT207380.1

HparOBP28-partial [*Holotrichia parallela*]. GenBank accession number KT207381.1

HparOBP29 [*Holotrichia parallela*]. GenBank accession number KT207382.1

HparOBP30-partial [*Holotrichia parallela*]. GenBank accession number KT207383.1

HparOBP31 [*Holotrichia parallela*]. GenBank accession number KT207384.1

HparOBP32 [*Holotrichia parallela*]. GenBank accession number KT207385.1

BhorOBP1 [*Batocera horsfieldi*]. GenBank accession number KC461118.1

BhorOBP2 [*Batocera horsfieldi*]. GenBank accession number KC461116.1

BhorOBP3 [*Batocera horsfieldi*]. GenBank accession number KC461117.1

BpraPBP [*Brachysternus prasinus*]. GenBank accession number AGG37860.1

PjapPBP-partial [*Popillia japonica*]. GenBank accession number AAC63436.1

EoriPBP-partial [*Exomla orientalis*]. GenBank accession number BAB70711.1

AcupPBP1-partial [*Anomala cuprea*]. GenBank accession number BAC06496.1

AschPBP1 [*Anomala schonfeldti*]. GenBank accession number BAF79599.1

AschPBP2-partial [*Anomala schonfeldti*]. GenBank accession number AB040983.1

AoctPBP-partial [*Anomala octiescostata*]. GenBank accession number BAC06499.1

HelePBP-partial [*Hylamorpha elegans*]. GenBank accession number KC676790.1

BhorPBP1 [*Batocera horsfieldi*]. GenBank accession number KM222577.1

BhorPBP2 [*Batocera horsfieldi*]. GenBank accession number KM222578.1

HparPBP1-partial [*Holotrichia parallela*]. GenBank accession number GQ395804.1

ArufPBP2-partial [*Anomla rufocuprea*]. GenBank accession number AB040978.1

ArufPBP3-partial [*Anomla rufocuprea*]. GenBank accession number AB040979.1

CbuqPBP1 [*Cyrtotrachelus buqueti*]. GenBank accession number KU845733.1

TcasOBPC1 [*Tribolium castaneum*]. GenBank accession number EFA07544.1

TcasOBPC2 [*Tribolium castaneum*]. GenBank accession number XM015982021.1

TcasOBPC4 [*Tribolium castaneum*]. GenBank accession number EFA07430.1

TcasOBPC9 [*Tribolium castaneum*]. GenBank accession number XM970593.4

TcasOBPC11 [*Tribolium castaneum*]. GenBank accession number XM962706.4

TcasOBPC12 [*Tribolium castaneum*]. GenBank accession number XM962784.4

TcasOBPC14 [*Tribolium castaneum*]. GenBank accession number XM965238.3

TcasOBPC15 [*Tribolium castaneum*]. GenBank accession number XM015979334.1

TcasOBPC16 [*Tribolium castaneum*]. GenBank accession number NM001143903.1

TcasOBPC17 [*Tribolium castaneum*]. GenBank accession number NM001143902.1

TcasOBPC18 [*Tribolium castaneum*]. GenBank accession number XM001812926.2

TcasOBPC21 [*Tribolium castaneum*]. GenBank accession number XM001813613.3

BhorOBPC1 [*Batocera horsfieldi*]. GenBank accession number GU575294.1

BhorOBPC2 [*Batocera horsfieldi*]. GenBank accession number GU575295.1

BhorOBPC3 [*Batocera horsfieldi*]. GenBank accession number GU584933.1

BhorOBPC4 [*Batocera horsfieldi*]. GenBank accession number GU584934.1
