# Supplementary material for: Transcriptome analysis in different developmental stages of Batocera horsfieldi (Coleoptera: Cerambycidae) and comparison of candidate olfactory genes
Source: PLoS One. 2018 Feb 23;13(2):e0192730. doi: 10.1371/journal.pone.0192730 (PMC5825065; doi:10.1371/journal.pone.0192730)
Supplement: S25 Text — (DOCX) [file pone.0192730.s025.docx]

HparCSP16 [*Holotrichia parallela*]. GenBank accession number AKI84399.1

HparCSP15 [*Holotrichia parallela*]. GenBank accession number AKI84398.1

HparCSP14 [*Holotrichia parallela*]. GenBank accession number AKI84397.1

HparCSP13 [*Holotrichia parallela*]. GenBank accession number AKI84396.1

HparCSP12 [*Holotrichia parallela*]. GenBank accession number AKI84395.1

HparCSP11 [*Holotrichia parallela*]. GenBank accession number AKI84394.1

HparCSP10 [*Holotrichia parallela*]. GenBank accession number AKI84393.1

HparCSP9 [*Holotrichia parallela*]. GenBank accession number AKI84392.1

HparCSP8 [*Holotrichia parallela*]. GenBank accession number AKI84391.1

HparCSP7 [*Holotrichia parallela*]. GenBank accession number AKI84390.1

HparCSP6 [*Holotrichia parallela*]. GenBank accession number AKI84389.1

HparCSP5-partial [*Holotrichia parallela*]. GenBank accession number AKI84388.1

HparCSP4 [*Holotrichia parallela*]. GenBank accession number AKI84387.1

HparCSP3 [*Holotrichia parallela*]. GenBank accession number AKI84386.1

HparCSP2 [*Holotrichia parallela*]. GenBank accession number AKI84385.1

HparCSP1 [*Holotrichia parallela*]. GenBank accession number AKI84384.1

TmolCSP1 [*Tenebrio molitor*]. GenBank accession number KP296743.1

TmolCSP2 [*Tenebrio molitor*]. GenBank accession number KP296744.1

TmolCSP3 [*Tenebrio molitor*]. GenBank accession number KP296745.1

TmolCSP4 [*Tenebrio molitor*]. GenBank accession number KP296746.1

TmolCSP5 [*Tenebrio molitor*]. GenBank accession number KP296747.1

TmolCSP6 [*Tenebrio molitor*]. GenBank accession number KP296748.1

TmolCSP7 [*Tenebrio molitor*]. GenBank accession number KP296749.1

TmolCSP8 [*Tenebrio molitor*]. GenBank accession number KP296750.1

TmolCSP9 [*Tenebrio molitor*]. GenBank accession number KP296751.1

TmolCSP10 [*Tenebrio molitor*]. GenBank accession number KP296752.1

TmolCSP11 [*Tenebrio molitor*]. GenBank accession number KP296753.1

TmolCSP12 [*Tenebrio molitor*]. GenBank accession number KP296754.1

ItypCSP1 [*Ips typographus*]. GenBank accession number GACR01000074.1

ItypCSP2 [*Ips typographus*]. GenBank accession number GACR01000075.1

ItypCSP4 [*Ips typographus*]. GenBank accession number GACR01000076.1

ItypCSP5 [*Ips typographus*]. GenBank accession number GACR01000077.1

ItypCSP6 [*Ips typographus*]. GenBank accession number GACR01000078.1

DponCSP1 [*Dendroctonus ponderosae*]. GenBank accession number KC113413.1

DponCSP2 [*Dendroctonus ponderosae*]. GenBank accession number KC113424.1

DponCSP3 [*Dendroctonus ponderosae*]. GenBank accession number KC113412.1

DponCSP4 [*Dendroctonus ponderosae*]. GenBank accession number KP736131.1

DponCSP6 [*Dendroctonus ponderosae*]. GenBank accession number KC113414.1

DponCSP8 [*Dendroctonus ponderosae*]. GenBank accession number KC113416.1

DponCSP11 [*Dendroctonus ponderosae*]. GenBank accession number KC113415.1

TcasCSP1 [*Tribolium castaneum*]. GenBank accession number NM001045808.1

TcasCSP2 [*Tribolium castaneum*]. GenBank accession number NM001045812.1

TcasCSP4 [*Tribolium castaneum*]. GenBank accession number NM001045820.1

TcasCSP5 [*Tribolium castaneum*]. GenBank accession number NM001045822.1

TcasCSP6 [*Tribolium castaneum*]. GenBank accession number NM001045823.1

TcasCSP7 [*Tribolium castaneum*]. GenBank accession number NM001045824.1

TcasCSP8 [*Tribolium castaneum*]. GenBank accession number NM001045825.1

TcasCSP9 [*Tribolium castaneum*]. GenBank accession number NM001045818.1

TcasCSP10 [*Tribolium castaneum*]. GenBank accession number NM001045813.1

TcasCSP11 [*Tribolium castaneum*]. GenBank accession number NM001045814.1

TcasCSP12 [*Tribolium castaneum*]. GenBank accession number NM001045815.1

TcasCSP13 [*Tribolium castaneum*]. GenBank accession number NM001045816.1

TcasCSP14 [*Tribolium castaneum*]. GenBank accession number NM001045817.1

TcasCSP15 [*Tribolium castaneum*]. GenBank accession number NM001045826.1

TcasCSP16 [*Tribolium castaneum*]. GenBank accession number DQ855502.1

TcasCSP17 [*Tribolium castaneum*]. GenBank accession number NM001045819.1

TcasCSP18 [*Tribolium castaneum*]. GenBank accession number NM001045821.1

TcasCSP19 [*Tribolium castaneum*]. GenBank accession number NM001045811.1

TcasCSP20 [*Tribolium castaneum*]. GenBank accession number NM001045809.1

AcorCSP1 [*Anomala corpulenta*]. GenBank accession number KM251633.1

AcorCSP2 [*Anomala corpulenta*]. GenBank accession number KM251634.1

AcorCSP3 [*Anomala corpulenta*]. GenBank accession number KM251635.1

AcorCSP4 [*Anomala corpulenta*]. GenBank accession number KM251636.1

AcorCSP5 [*Anomala corpulenta*]. GenBank accession number KM251637.1

BhorCSP1 [*Batocera horsfieldi*]. GenBank accession number HQ587040.1

BhorCSP2 [*Batocera horsfieldi*]. GenBank accession number HQ587041.1

BhorCSP3 [*Batocera horsfieldi*]. GenBank accession number HQ587042.1

GdauCSP1 [*Galeruca daurica*]. GenBank accession number KY885471.1

GdauCSP2 [*Galeruca daurica*]. GenBank accession number KY885472.1

GdauCSP3 [*Galeruca daurica*]. GenBank accession number KY885473.1

GdauCSP4 [*Galeruca daurica*]. GenBank accession number KY885474.1

GdauCSP5 [*Galeruca daurica*]. GenBank accession number KY885475.1

GdauCSP6 [*Galeruca daurica*]. GenBank accession number KY885476.1

GdauCSP7 [*Galeruca daurica*]. GenBank accession number KY885477.1

GdauCSP8 [*Galeruca daurica*]. GenBank accession number KY885478.1

GdauCSP9 [*Galeruca daurica*]. GenBank accession number KY885479.1

GdauCSP10 [*Galeruca daurica*]. GenBank accession number KY885480.1

PaenCSP1 [*Pyrrhalta aenescens*]. GenBank accession number KX298777.1

PaenCSP2 [*Pyrrhalta aenescens*]. GenBank accession number KX298778.1

PaenCSP3 [*Pyrrhalta aenescens*]. GenBank accession number KX298781.1

PaenCSP4 [*Pyrrhalta aenescens*]. GenBank accession number KX298782.1

PaenCSP5 [*Pyrrhalta aenescens*]. GenBank accession number KX298783.1

PaenCSP6 [*Pyrrhalta aenescens*]. GenBank accession number KX298784.1

PaenCSP7 [*Pyrrhalta aenescens*]. GenBank accession number KX298785.1

PmacCSP1 [*Pyrrhalta maculicollis*]. GenBank accession number KX290641.1

PmacCSP2 [*Pyrrhalta maculicollis*]. GenBank accession number KX290643.1

PmacCSP3 [*Pyrrhalta maculicollis*]. GenBank accession number KX290645.1

PmacCSP4 [*Pyrrhalta maculicollis*]. GenBank accession number KX290646.1

PmacCSP5 [*Pyrrhalta maculicollis*]. GenBank accession number KX290647.1

PmacCSP6 [*Pyrrhalta maculicollis*]. GenBank accession number KX290649.1

PmacCSP7 [*Pyrrhalta maculicollis*]. GenBank accession number KX290650.1

PmacCSP8 [*Pyrrhalta maculicollis*]. GenBank accession number KX290642.1

PmacCSP9 [*Pyrrhalta maculicollis*]. GenBank accession number KX290644.1

PmacCSP10 [*Pyrrhalta maculicollis*]. GenBank accession number KX290648.1

DhelCSP1 [*Dastarcus helophoroides*]. GenBank accession number KF984186.1

DhelCSP2 [*Dastarcus helophoroides*]. GenBank accession number KF984187.1

DhelCSP3 [*Dastarcus helophoroides*]. GenBank accession number KF984188.1

DhelCSP4 [*Dastarcus helophoroides*]. GenBank accession number KF984189.1

DhelCSP5 [*Dastarcus helophoroides*]. GenBank accession number KF984190.1

DhelCSP6 [*Dastarcus helophoroides*]. GenBank accession number KF984191.1

DhelCSP7 [*Dastarcus helophoroides*]. GenBank accession number KF984165.1

CbowCSP1 [*Colaphellus bowringi*]. GenBank accession number KT381509.1

CbowCSP2 [*Colaphellus bowringi*]. GenBank accession number KT381510.1

CbowCSP3 [*Colaphellus bowringi*]. GenBank accession number KT381511.1

CbowCSP4 [*Colaphellus bowringi*]. GenBank accession number KT381512.1

CbowCSP5 [*Colaphellus bowringi*]. GenBank accession number KT381513.1

CbowCSP6 [*Colaphellus bowringi*]. GenBank accession number KT381514.1

CbowCSP7 [*Colaphellus bowringi*]. GenBank accession number KT381515.1

CbowCSP8 [*Colaphellus bowringi*]. GenBank accession number KT381516.1

CbowCSP9 [*Colaphellus bowringi*]. GenBank accession number KT381517.1

CbowCSP10 [*Colaphellus bowringi*]. GenBank accession number KT381518.1

CbowCSP11 [*Colaphellus bowringi*]. GenBank accession number KT381519.1

CbowCSP12 [*Colaphellus bowringi*]. GenBank accession number KT381520.1

MaltCSP1 [*Monochamus alternatus*]. GenBank accession number KF984158.1

MaltCSP2 [*Monochamus alternatus*]. GenBank accession number KF984159.1

MaltCSP3 [*Monochamus alternatus*]. GenBank accession number KF984160.1

MaltCSP4 [*Monochamus alternatus*]. GenBank accession number KF984161.1

MaltCSP5 [*Monochamus alternatus*]. GenBank accession number KF984162.1

MaltCSP6 [*Monochamus alternatus*]. GenBank accession number KF984163.1

MaltCSP7 [*Monochamus alternatus*]. GenBank accession number KF984164.1

MaltCSP8 [*Monochamus alternatus*]. GenBank accession number KF984157.1

MaltCSP9 [*Monochamus alternatus*]. GenBank accession number KF984162.1

MaltCSP10 [*Monochamus alternatus*]. GenBank accession number KJ027563.1

MaltCSP11 [*Monochamus alternatus*]. GenBank accession number KJ027564.1

MaltCSP12 [*Monochamus alternatus*]. GenBank accession number KJ027565.1

RdomCSP1 [*Rhyzopertha dominica*]. GenBank accession number KJ186797.1

RdomCSP2 [*Rhyzopertha dominica*]. GenBank accession number KJ186798.1

RdomCSP3 [*Rhyzopertha dominica*]. GenBank accession number KJ186799.1

RdomCSP4 [*Rhyzopertha dominica*]. GenBank accession number KJ186800.1

RdomCSP5 [*Rhyzopertha dominica*]. GenBank accession number KJ186801.1

RdomCSP6 [*Rhyzopertha dominica*]. GenBank accession number KJ186802.1

RdomCSP7 [*Rhyzopertha dominica*]. GenBank accession number KJ186803.1

RdomCSP8 [*Rhyzopertha dominica*]. GenBank accession number KJ186804.1

HoblCSP1 [*Holotrichia oblita*]. GenBank accession number HQ683720.1

HoblCSP2 [*Holotrichia oblita*]. GenBank accession number HQ688991.1

LoryCSP3 [*Lissorhoptrus oryzophilus*]. GenBank accession number KF410819.1

LoryCSP6 [*Lissorhoptrus oryzophilus*]. GenBank accession number KF410820.1

LoryCSP8 [*Lissorhoptrus oryzophilus*]. GenBank accession number KF410821.1

LoryCSP9 [*Lissorhoptrus oryzophilus*]. GenBank accession number KF410822.1

LoryCSP10 [*Lissorhoptrus oryzophilus*]. GenBank accession number KF410823.1

RferCSP [*Rhynchophorus ferrugineus*]. GenBank accession number KT748773.1
