# Supplementary material for: Transcriptome analysis in different developmental stages of Batocera horsfieldi (Coleoptera: Cerambycidae) and comparison of candidate olfactory genes
Source: PLoS One. 2018 Feb 23;13(2):e0192730. doi: 10.1371/journal.pone.0192730 (PMC5825065; doi:10.1371/journal.pone.0192730)
Supplement: S2 Table — (DOCX) [file pone.0192730.s027.docx]

| >*Cluster-8309.27359* BhorOBP1  AGAAGCTGGCGGAAACCGTGTGAGCATTATGTGTATCGTTTGCAGATAGTGAACGTCAAG  GAATATCTGAAGAACATGAAGGTCCTCGTCGGTTTGTTAGTTGCCTGCGTGGTGGTTTCT  CTATCACAGGCCGACCTTATCATAACCGATGCTCAAAAGGCCAAGCTACTGGCCCACCAC  AAAGTGTGTTCGGGTGAGGTGAACGTGAAGGACGACGTTGTAGAGAAGCTCCTCGATGGC  GTCTTCAGCGACGACCAGGTCTTCAAGGACTATCTCCTTTGCTTGTCCAAAAGGATTGGC  TTCCAAGACGAGGCTGGCAAAGTGCAAAAGGATGTCATCATCGCCAAACTGAAAGATTCG  GTCCAAGACCCTTCCAAGGCGGAGGAGTACACCGAGAAGTGTCTCGTAGAAGAGGGAAAG  CCCGCTGACGTGGTTTATAAAGTCGTCACTTGTTTACAGGAGAACACGCCAAATGTCTTG  TTTGCTTAG |  |  |
| --- | --- | --- |

>*Cluster-8309.40672* BhorOBP2

AAGACTCGCTTGGAAGAGGAAAAGGAGAAACTGCACGAAATCCACGAGGAGTGCCAAGCAGACCCGGACACCTACGCCGACGACGCACTCCTAGAGAACCTGTCCGAAAACTTGGACAATCCTCAAGTCGAGGCGCACATGCTCTGCGAGTCCACCAAAGTTGGCCTGCAAAGCGCGGACGGTGATCTAGATTTGGACACCATCAGGACCAAAGTAGCTCTATCCGTGACGGACAGCGCGAAAGTCGACCGTCTGGTCAAGGAGTGCGCCATCAAGAAGAAGAGTCCCGAGAAGACCGCTATTCACCTGTTCTTGTGTTTGGATAAGAATGGCGTCACTTATTTCCACGAATTTTAG

>*Cluster-8309.41624* BhorOBP3

AAGTTGATAGCAAACATGAAAATCGTTCTTGTGTTGTTGTGTACTATCGTCGGCATCTGGGTGAGTCACATGTGA

>*Cluster-8309.12499* BhorCSP1

AAAAAAAAAAAAAAAAAAACGTCTTCGGCGATCGTATCGAAGTTCTTCATCATGCAACCG

GTAGTTGTCTTACTTCTTGGTGCTCTCTGTGTCTTTGCGGACGCTGACTCTCACTACACG

ACCAAGTACGACAATATTGATTTGGACGAGATCCTTCGCAATGAGAGGCTGCTGAAGAAC

TACTACCAGTGTCTCGAAGGCACGAAATCGTGCACAAAGGACGGCCAAGAGCTGAAAGAC

ATCCTGCCAGATGCGCTCAAAACCAATTGTTCCAAATGTAACGAGATTCAGAAGAATGGA

GCCGAAAAAGTCATCCGTTACTTGATCGATAACAAAGAGGATTGGTTCAAGAATCTCGAA

GCCATCTACGATCCAGAAAACGTATATAGAAAACAATTGGAACAAGAAGCTCAACAAAGG

GGTATTAAAATACCATAG

>*Cluster-8309.41714* BhorCSP2

ATGAACCGATTCTTGCTGATCCTGTCCGTGGCCCTAATCGGCGTGGTGGCCGGCCAGAAGTACACCACCAAGTACGACAACATCGATCTGGATCAGATCATCAAGAGCGACAGACTGTTGAAGAACTACGTCGACTGCGTCTTGGAACGTGGAAATTGCACTCCAGACGGACAAGAGCTCAAGAAGAATATTCCCGATGCGCTTTTGACAGACTGCAGCAAGTGTAGCGACAXX

>*Cluster-8309.54261* BhorCSP3

TTTACGATGGTACGTTTCCTTCCTGTGTTGGTGGTCGCCGGCGCCGTACTGGGGCTGATC

GACTCTGCCGCCGGCCAGTACTACGCCTCCAAGTACGACCACATCGACGTCGACGCTATC

CTGAATAACAGGAGGATCGTGAACTACTATGCCGCCTGCCTGTTGTCGAAAGGGCCTTGT

CCTCCGGAAGGAGTGGACTTCAAACGGATTTTACCCGAAGCCCTGAGAACCAACTGCCGC

AGGTGCACCGAAAAACAAAAAACCGTTACCCTCCGGGCCATCAGACGCCTGAAAAAGGAG

TACCCCAAGGTGTGGGCCCAACTAGAAGAACAGTGGGACCCCGACCGCAGCTACATATCC

AAATTCGAATCGACCTTCGGCGCCAAACCGTCCGAATCCACCGCGCAATCCGTACAAATA

GTAAACAGGTTCGCTTCTCCGGAAAACGACAACAAGACCATAGACAATACCATAAGCCGG

ATGGCTACCACTTCTTCGCCTACAACTACTAGTACCAGTACAGTTGGCAGTACCGTTTCG

AGTACAACCACTAGCAGACCCACACCCACAAAAACCACAACCGCAACCACCACCACAACC

ACCACAGTTAGTACTAAAGCTGTTCCCGTAACAAAAGCTACTACTCGTAGAGTTAGTATC

ATAAACACCATCATCAAACAAAACATTAACACCATCTCAGCTACCGCTAAACCCACGAAG

CGTCCCAGACCGAGCATAGGACAGAGCATACAGGCGACGGTGGAAGTAGTGAAATCTATA

GAGAAAATGGTTAATCAGATAGCACGGGAAAAATTGGGATTCATTAGTCGCCTACTCATC

GGATAA

>*Cluster-8309.39777* BhorOBPC1

AGCAATATGAAAACTGTGTTTGTGGTGTGTCTCTTGGTCGCACTAGCGGCTTCAGATGCC

GACATAGAGAAAAAAATTCAAGAATGTGATAAGGAAACGGGCTCAACACTATCAGAAGTC

ACGAAATATCTTCTTGCCGACGACACTAAGAACGACGAAAAAGCGACTAAACACATAATG

TGTATGTTCAAACAACACGGAGCCATCGACGGCGAAGGCCATCTCGATATGGAGAAGATT

CGACTATCGGTAAACAACTACATGAAGACGATAGATGCCGCCGACGACAAAAAGGCCTTG

GAGTGCGTCAAGGAGAAGGACACGGCCGAAGAAACGGCCCTCGCAGTCGGCAAGTGCGTG

GAGAAAAGAAGAGCTGAACTTACTAGCTCAAAGTAA

>*Cluster-8309.59754* BhorOBPC2

CTTGTTAAAATGAATAGTGTAATATGTTTAGTCGTGGCTTCTGTTCTAGCGTCTACAGTT

CACGCTATTTTCGACCAATCAAAATTCGGTCCCAAGCTTCAGGAATTAGCTAAAAATCTT

GGCTCTACGTGTAGATCGAAATCGGGTACGGACCAAGCTTCCATTGATAAAGTAATAAAT

GGGGAATTTATTGACGAACCGAAAATAAAGGTTTACATGAGATGTCTTTTCACGGAAAGT

GGAGTGATCACCGAAAAAGGTTTGAACCTGGAACTCGTGGCTCAATTACTGCCACCCGAA

ATTACAGATGAGTCTATAAAGAATGCGAAGATCTGTCTCGGAAAAATAAAAGGAATCACA

AATGTAGAAGAGAGGGCTTTCAATTTCTTTAAGTGTTATTACAGCCAAAATTCTGATCTT

TTCGTTTTCTTCTAA

>*Cluster-8309.47478* BhorOBPC3

ATGAACAAGTTGACTGCGGTCTTCTCTGGAGTCTCCTTCTTCACAGCGCACTCTCTGACCAATCTTTCAACTTTGGCCTTATCGCTGACGGTGAGGGAGATCTTCXTGTTTGATGACTTTCAAGTCCAAATCACCGTTGGGCTTCTGGAGGCCGACAGCTTTGGATTCGCAGAGCATGTGGGCGCCGACTTTGGGGTTGTTGATGTTTGCGGCGAGGTTGTGCAACAAATGATGGTCGACATGGGTGGCTGGGTCGGCTTGGCATTTGTCGTGGATTTCCTGGAGTTTCXX

>*Cluster-8309.31830* BhorOBPC1

GTTTCTGTTTCGATCTTCCACAAGAAGCAACAACAGTTGGACAAAATGAAAGCCGTATTC

GTTTTTGCTTGCGTCGTCGTTGCAGCTTTGGCCGCCAGCCTTTCCGAAGAGGAGAAAAAA

CTCCAGGAAATCCACGACAAATGCCAAGCCGACCCAGCCACCTACGTCGACCACGAATTG

TTGCACAACCTCTCCGCAAACATCAACAACCCCAAAGTCGGCGCCCACATGCTCTGCGAA

TCCAAAGCTGTCGGTCTCCAGAAGCCAAACGGCGAATTGGACTTGAAAGTCATCAAGCAG

AAGATCTCCCTCACCGTCAGCGATAAGGCCAAAGTTGAAAGATTGGTTAGAGAATGCGCT

GTGAAGAAGCAGACCCCAGAGAAGACTGCTGTTAACCTATTCATGTGTTTGGACAAAGAT

GGAGTCACATACTTCCATGAATTCTAA
