# Supplementary material for: Transcriptome analysis in different developmental stages of Batocera horsfieldi (Coleoptera: Cerambycidae) and comparison of candidate olfactory genes
Source: PLoS One. 2018 Feb 23;13(2):e0192730. doi: 10.1371/journal.pone.0192730 (PMC5825065; doi:10.1371/journal.pone.0192730)
Supplement: S3 Table — (DOCX) [file pone.0192730.s028.docx]

| Gene |  | 5'--3' |
| --- | --- | --- |
| *18S* | F | GAGACTCTAGCCTGCTAACT |
|  | R | TGTTTGTACGCCGACAGT |
| *BhorOBP1* | F | TAGATGCCATGCATAGAGGT |
|  | R | GCCAATACTAGGTGGAGCAT |
| *BhorOBP2* | F | CAGAGGACGACCACATAGCAT |
|  | R | TCGTCGTGTGGATTGTTTGCT |
| *BhorOBP3* | F | TGCTCAGAGTCTGTGGGACT |
|  | R | TCGGAACTGGGTTCTCTCGT |
| *BhorCSP1* | F | CATCGATCTGGACGAGATCCT |
|  | R | TGCCCTTAAGACAGTCCACGT |
| *BhorCSP2* | F | TGATCGACAACAAGGCCGAT |
|  | R | TCGTATTTAGCCTCGAGTTCCT |
| *BhorCSP3* | F | TAGACAGGGGAGGTTGCACTGCT |
|  | R | TCCCACATGTCCGCCTTGTTGT |
| *BhorOBP C1* | F | TATGGAGAAGGTCCGACTGT |
|  | R | TGTCCTTCTCCTCGACGCACT |
| *BhorOBP C2* | F | TAGTAGTGGTGTCTTCGCTCT |
|  | R | CGAGTCCACTAATTCCAGAAGT |
| *BhorOBP C3* | F | TGGAGCACATCCACGACGAGT |
|  | R | TGGTGGACTCGCAGAGCATGT |
| *BhorOBP C4* | F | TGGACTTGAATGTCATCAAGCA |
|  | R | TGCTTCTTCACAGCGCATTCT |
